# Quantum-Chemistry Study of the Photophysical Properties of 4-Thiouracil and Comparisons with 2-Thiouracil

**DOI:** 10.1021/acs.jpca.3c06310

**Published:** 2024-03-20

**Authors:** Miriam Navarrete-Miguel, Angelo Giussani, Mercedes Rubio, Martial Boggio-Pasqua, Antonio Carlos Borin, Daniel Roca-Sanjuán

**Affiliations:** † Instituto de Ciencia Molecular, Universitat de València, P.O. Box 22085, ES-46071 Valencia, Spain; ‡ Departament de Química Física, Universitat de València, 46100 Burjassot, Spain; § Laboratoire de Chimie et Physique Quantiques, IRSAMC, CNRS et Université Toulouse 3, 118 route de Narbonne, 31062 Toulouse, France; ∥ Department of Fundamental Chemistry, Institute of Chemistry, University of São Paulo, Av. Prof. Lineu Prestes, 748, São Paulo CEP 05508-000, Brazil

## Abstract

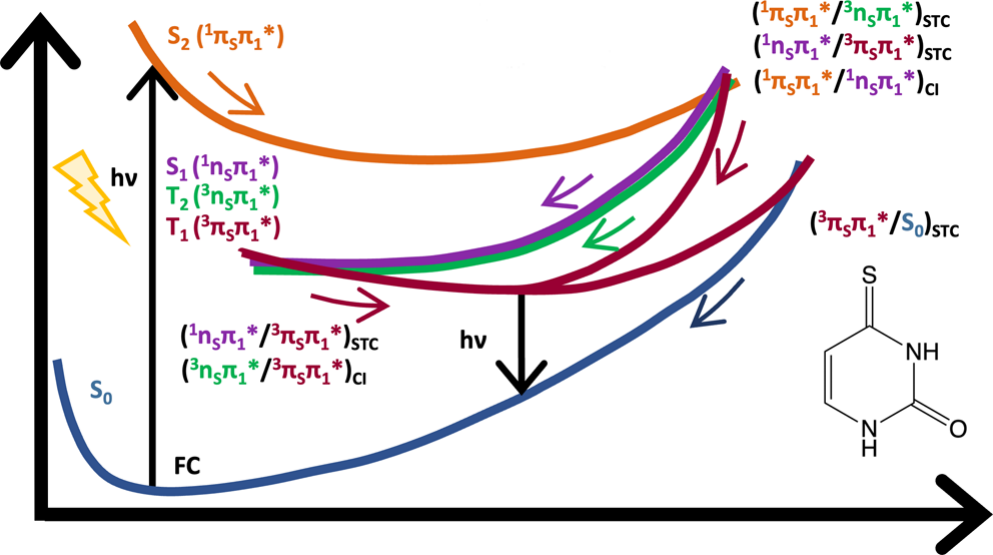

DNA in living beings is constantly damaged by exogenous and endogenous agents. However, in some cases, DNA photodamage can have interesting applications, as it happens in photodynamic therapy. In this work, the current knowledge on the photophysics of 4-thiouracil has been extended by further quantum-chemistry studies to improve the agreement between theory and experiments, to better understand the differences with 2-thiouracil, and, last but not least, to verify its usefulness as a photosensitizer for photodynamic therapy. This study has been carried out by determining the most favorable deactivation paths of UV–vis photoexcited 4-thiouracil by means of the photochemical reaction path approach and an efficient combination of the complete-active-space second-order perturbation theory//complete-active-space self-consistent field (CASPT2//CASSCF), (CASPT2//CASPT2), time-dependent density functional theory (TDDFT), and spin-flip TDDFT (SF-TDDFT) methodologies. By comparing the data computed herein for both 4-thiouracil and 2-thiouracil, a rationale is provided on the relatively higher yields of intersystem crossing, triplet lifetime and singlet oxygen production of 4-thiouracil, and the relatively higher yield of phosphorescence of 2-thiouracil.

## Introduction

Chemically modified nucleobases have been used in different applications, from chemotherapy^1−4^ and fluorescence markers^5−14^ to nanomaterials.^15−18^ Among them, thiated nucleobases (thiobases or thionucleobases) have received considerable attention due to their potential applications in photodynamic therapy.^19−25^ Thionucleobases differ from the canonical nucleobases because one(or more) oxygen atom(s) is(are) replaced by a sulfur atom(s), which greatly increases the intersystem crossing yields, conferring them the characteristic of being species with very high quantum yield for triplet state population, which can be employed to stimulate in situ generation of reactive singlet oxygen (^1^O_2_). This excited state of molecular oxygen is a toxic species employed in photodynamic cancer therapy to damage biomolecules and produce cell death. ^1^O_2_ is generated when a photosensitizer (photodynamic drugs), after being exposed to a specific wavelength and excited to a triplet excited state, deactivates to the ground state and the energy released is transferred to the oxygen in the ground state (^3^O_2_). The photosensitizer is a key component in this therapy, and therefore, most of the research focuses on the design of more efficient photosensitizers and understanding their physicochemical and medical applications.^26−28^ Thionucleobases are prospective photosensitizers.

**Figure 1:**
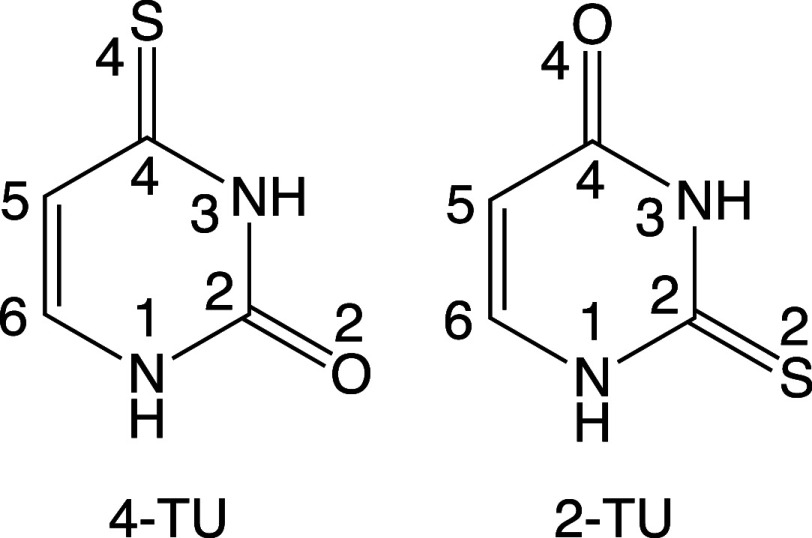
Chemical structure and labeling numbering for 4-TU and 2-TU.

Among thionucleobases, 2-thiouracil (2-TU) (Figure 1, right) is a very interesting species because of its high triplet quantum yield (Φ_ISC_ = 0.75 ± 0.20),^29^ high phosphorescence yield (Φ_Ph_ = 0.65–0.70),^30,31^ and short triplet lifetime (τ = 0.07 ± 0.02 μs).^32^ As it has a limited absorption at 355 nm, no attempt was made to measure its singlet oxygen yield,^33^ but it has been supposed to be similar to that observed for 2-thiothymine (Φ_Δ_ = 0.36 ± 0.02).^29^

2-TU deactivation pathways have been investigated computationally by different authors. Cui and Fang,^34^ by employing the complete-active-space second-order perturbation theory//complete-active-space self-consistent field (CASPT2//CASSCF) protocol and imposing *C*_S_ symmetry in most calculations, were the first to propose three different deactivation mechanisms (S_2_^1^(π_S_π_1_*) → S_1_^1^(n_S_π_1_*) → T_1_^3^(π_S_π_1_*), S_2_^1^(π_S_π_1_*) → T_2_^3^(n_S_π_1_*) → T_1_^3^(π_S_π_1_*), and S_2_^1^(π_S_π_1_*) → T_3_^3^(n_S_π_1_*) → T_2_^3^(n_S_π_1_*) → T_1_^3^(π_S_π_1_*)). However, these mechanisms are compromised due to symmetry constraints imposed by the authors. Later on, Gobbo and Borin^35^ employed the same computational protocol, but without symmetry constraints, and proposed two similar deactivation pathways (S_2_^1^(π_S_π*) → S_1_^1^(n_S_π*) → T_1_^3^(π_S_π*) and S_2_^1^(π_S_π*) → T_2_^3^(n_S_π*) → T_1_^3^(π_S_π*)). Shortly afterward, Mai et al.,^36^ employing several levels of theory (CASSCF, MRCIS—multireference configuration interaction including single excitations, SS-CASPT2—state-specific CASPT2, and MS-CASPT2—multistate CASPT2), optimized minima and crossing points, obtaining strongly distorted geometries, and proposed new photophysical deactivation mechanisms as, for instance, the S_2_ → S_1_ → T_2_ → T_1_ sequence of population transfers. They also reported a spin–orbit coupling (SOC) between the S_1_ and T_2_ states of about 150 cm^–1^, even though the S_1_ and T_2_ states are of the same n_S_π* nature, which is in apparent contradiction to El-Sayed’s rule;^37^ nonetheless, they detected in the photodynamics that the T_2_^3^(n_S_π*) and T_1_^3^(ππ*) states reversed their energetic order temporarily, consequently determining that the above-reported S_2_ → S_1_ → T_2_/T_1_ pathway can be represented as ^1^π_S_π* → ^1^n_S_π* → ^3^π_S_π* decay, where the electronic states are labeled by their nature.^38^ Consequently, from now on we will refer to the deactivation path of this molecule as S_2_^1^(π_S_π*) → S_1_^1^(n_S_π*) → T_2_/T_1_^3^(π_S_π*).

4-Thiouracil (4-TU) (Figure 1, left) is another important thionucleobase but less explored computationally. Its absorption spectrum is characterized by a strong band in the UVA region, with a maximum of around 330 nm, and by a weaker one in the UVB region, at 240 nm.^39^ Its lowest-lying triplet state is of ππ* nature (T_1_^3^(ππ*)), while T_2_ is of nπ* character (T_2_^3^(nπ*)). In comparison to 2-TU, it has a higher (near unity) triplet population quantum yield (Φ_ISC_ = 0.90 ± 0.15),^33,39,40^ with the highest singlet oxygen formation yield (Φ_Δ_ = 0.49 ± 0.02)^33,41^ among thiobases. At 77 K, 4-TU shows a low phosphorescence yield (Φ_Ph_ = 0.15).^42^ Finally, a triplet lifetime of τ = 0.23 ± 0.02 μs was measured in anaerobic conditions and τ = 0.17 ± 0.02 μs in aerobic conditions.^39^ A more recent study on 4-TU^43^ has shown, by means of sub-30 fs broadband transient absorption spectroscopy in the UV with state-of-the-art QM/MM simulations, that its most relevant decay paths are S_2_^1^(π_S_π*) → S_1_^1^(n_S_π*) → T_1_^3^(π_S_π*) and S_2_^1^(π_S_π*) → T_2_^3^(n_S_π*) → T_1_^3^(π_S_π*), with the former being the most probable. The study also explained why populating the triplet state of 4-TU is two times faster than that of 2-TU. This is attributed to the planar geometry of 4-TU in contrast to 2-TU and the fact that, in 4-TU, the minimum energy region of the singlet nπ* state crosses the potential energy hypersurface of the triplet state of ππ* nature enhancing the possibility of population transfer to the triplet state, whereas 2-TU needs to overcome a barrier of ca. 0.1 eV in order to populate the triplet manifold. However, a detailed knowledge about the factors influencing the experimentally observed phosphorescence yields and triplet lifetimes was still missing. Therefore, the present contribution is devoted to providing deeper details on the intrinsic photophysical properties of the 4-TU molecule by using the SS-CASPT2 method and different flavors of the density functional theory (DFT) method with the B3LYP functional. Furthermore, by comparing the photophysical properties and the geometry differences between 4-TU and 2-TU, we achieve a more comprehensive interpretation of their behavior and characteristics.

## Computational Details

Geometry optimizations of excited-state minima, conical intersections (CIs), singlet–triplet crossings (STCs), and minimum energy paths (MEPs) have been mainly performed with the CASPT2//CASSCF protocol, which consists of geometry optimizations at the CASSCF^44^ level, followed by single-point energy calculations at the state-specific CASPT2^45^ (SS-CASPT2; here after CASPT2) level on top of the converged geometries. Calculations were performed without symmetry constraints and the ANO-S-VDZP basis set, since the CASPT2 method does not depend much on the basis set’s size in the case of thiouracils^46^ and also in larger systems.^47^ However, in order to test this dependence on the basis set, CASPT2 benchmark calculations were performed in this study with the ANO-S-VDZP, ANO-L-VDZP, and ANO-L-VTZP basis sets (see Tables S1–S5 on the Supporting Information), obtaining the same conclusions for energies and SOCs. CASSCF and CASPT2 calculations were carried out with MOLCAS 8 software.^48^ CIs and STCs were computed as the minimum energy crossing points (MECPs) on the singlet–singlet, triplet–triplet, and singlet–triplet manifolds obtained with the restricted Lagrange multiplier technique,^49^ imposing the constraint of degeneracy between the two states considered. In order to perform a geometrical analysis and comparison, some specific geometry optimizations (indicated in Results and Discussion) were also carried out: (1) at the CASPT2/ANO-S-VDZP level and (2) with the spin-flip time-dependent DFT (SF-TDDFT),^50^ (3) time-dependent DFT (TDDFT), and/or (4) Tamm–Dancoff DFT approach (TDA)^51^ methods, using the B3LYP functional and the 6-311 + G** basis set, with Q-Chem 5 software.^52^

MEPs were computed with a modified version of the steepest descendent algorithm developed by Anglada and Boffil, as proposed by Müller-Brown.^53^ Mass-weighted coordinates (in bohr (amu)^1/2^) were used, which means that they are equivalent to the so-called intrinsic reaction coordinate (IRC).

**Figure 2:**
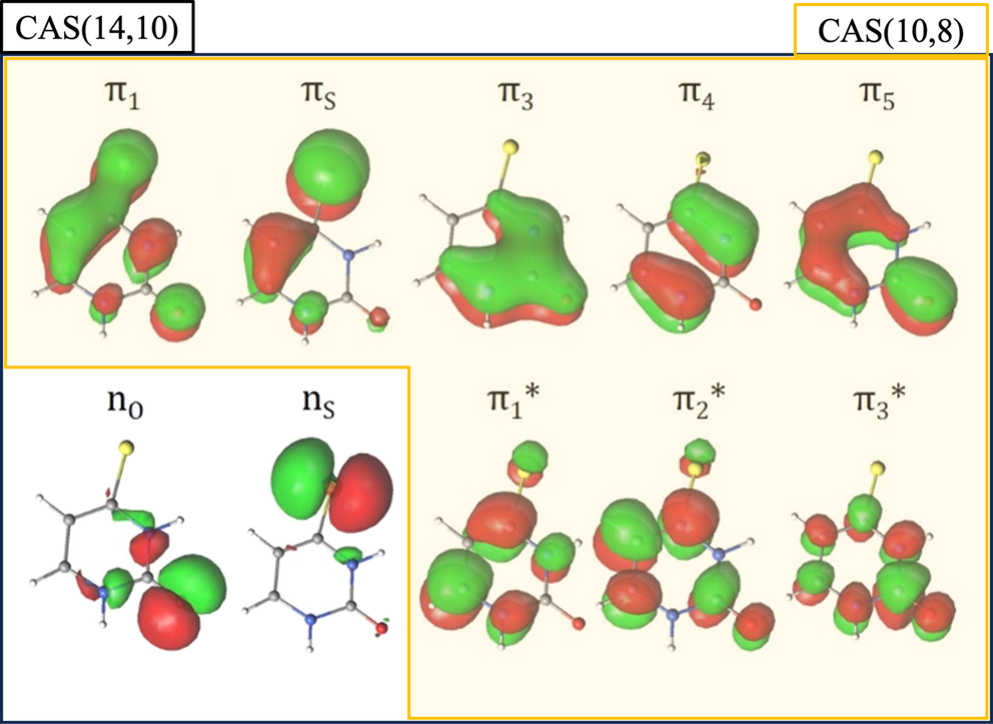
Valence natural orbitals computed at the SA(7)-CASSCF(14,10)/ANO-S-VDZP (highlighted in black) and SS-CASSCF(10,8)/ANO-S-VDZP (highlighted in yellow) levels of theory at the ground-state equilibrium geometry for 4-thiouracil.

The CASSCF active space for ground state (GS) geometry optimization comprises the π and π* orbitals and electrons (CASSCF(10,8)) only (Figure 2, highlighted in yellow). For this geometry optimization, state-specific CASSCF wave functions were employed; that is, a single root was employed in the CASSCF step. S_0_/T_1_ MECP geometry optimization was obtained at the CASPT2 level due to the differential correlation initially detected when performing CASSCF geometry optimization of this point. This CASPT2 optimization was performed with an active space of CAS(10,8) including the π-system orbitals. Remaining geometry optimizations were carried out with a larger active space, including 14 electrons distributed over 10 molecular orbitals (MOs; 8 π and π* orbitals, plus the oxygen and sulfur lone pairs; CASSCF(14,10)) (Figure 2, highlighted in black), including the necessary roots to consider the desired states.

In order to add dynamical correlation effects to all of the geometries corresponding to the previously optimized minima, crossing points, and MEP points, the CASPT2 method^49,53,54^ was used with an active space that includes all π, π* and lone pair orbitals, that is, CASSCF(14,10). Seven singlets and seven triplets were computed using as zeroth-order reference for each spin multiplicity a state-average CASSCF (SA-CASSCF) wave function (with equal weights for each state). The zeroth-order Hamiltonian with an IPEA^55^ value of zero and an imaginary level shift^56^ of 0.2 au have been used in CASPT2 calculations. Reference weights of the CASSCF wave functions in the CASPT2 computations for each root are compiled in Table S6, indicating that it does not include intruder states. Associated oscillator strengths (*f*) were computed using transition dipole moments (length representation) obtained with the CAS state interaction (CASSI) method^57^ and state energy differences calculated at the CASPT2 level as described above.

Benchmark computations were conducted on the different minima and the MECPs by comparing CASPT2//CASSCF and MS-CASPT2//CASSCF protocols (see Tables S7 and S8 in the Supporting Information). The outcomes consistently offer a comparable perspective in both scenarios, with the SS-CASPT2 values establishing a lower limit of 0.01 eV for the energy difference between the states on the degeneracy regions, and MS-CASPT2 values establishing an upper limit of 0.3 eV in these points where we would also expect degeneration. The use of CASSCF for geometries and CASPT2 for the final energies also introduces a certain degree of differential correlation specially in the crossing points. However, almost degeneracies are kept when recomputing energies at the CASPT2 level on top of the CASSCF structures with gaps lower than 0.2 eV. All of these differences are not too high and allow providing a reliable description with the CASPT2//CASSCF protocol.

## Results and Discussion

### Franck–Condon Region Spectroscopy

The CASPT2 vertical absorption energies (*E*_v_^abs^) computed at the CASSCF ground state optimized geometry (FC region), corresponding nature (CSF), *f* values, dipole moments (μ), and weight of the most important configurations in the CASSCF wave function for the seven lowest-lying singlet and triplet electronic states of 4-TU are collected in Table 1. We also added our computed data at TDDFT and TDA levels, and the data obtained by Borrego-Varillas et al.^43^

**Table 1:** 4-Thiouracil Vertical Absorption Energies (*E*_v_^abs^, eV), Oscillator Strengths (*f*), Nature and Weights (%) of the Main Excitations, and Dipole Moments (μ, Debye) for the Seven Lowest Singlet and Triplet States at the Franck–Condon Region at Different Levels of Theory Data from Ref (43) and Experimental Studies are also Provided, Both Obtained in Water Solution (cs = Closed Shell, de = Double Excitation)

CASPT2						TDDFT					TDA						ref (43)		exp. data	
state	*E*_v_^abs^	*f*	nature	CASSCF weight	μ	state	*E*_v_^abs^	*f*	nature	weight	state	*E*_v_^abs^	*f*	nature	weight	state	*E*_v_^abs^	*f*	*E*_v_^abs39^	*E*_v_^abs41^
S_0_			cs	78	4.28	S_0_					S_0_									
			^1^(π_S_π_1_*)	11																
T_1_	2.59		^3^(π_S_π_1_*)	57	3.02	T_1_	2.38	0.000	^3^(π_S_π_1_*)	50	T_1_	2.49	0.000	^3^(π_S_π_1_*)	49					
			^3^(π_1_π_1_*)	30																
T_2_	2.62		^3^(n_S_π_1_*)	86	2.87	T_2_	2.48	0.000	^3^(n_S_π_1_*)	49	T_2_	2.55	0.000	^3^(n_S_π_1_*)	49					
S_1_	2.75	0.000	^1^(n_S_π_1_*)	88	3.10	S_1_	2.79	0.000	^1^(n_S_π_1_*)	50	S_1_	2.80	0.000	^1^(n_S_π_1_*)	50	S_1_^1^(n_S_π_1_*)	2.82	0.00		
T_3_	3.84		^3^(π_S_π_2_*)	34	3.64	T_3_	3.78	0.000	^3^(π_1_π_1_*)	39	T_3_	3.88	0.000	^3^(π_1_π_1_*)	41					
			^3^(π_1_π_1_*)	33					^3^(π_S_π_2_*)	6.9										
S_2_	4.00	0.419	^1^(π_S_π_1_*)	47	4.55	S_2_	4.22	0.298	^1^(π_S_π_1_*)	46	S_2_	4.49	0.347	^1^(π_S_π_1_*)	37	S_2_^1^(π_S_π_1_*)	3.73	0.43	3.79	3.78
			^1^(π_1_π_1_*)	18																
			de ^1^(2π_S_π_1_*)	3																
			de ^1^(π_S_π_1_*+ π_S_π_2_*)	2																
S_3_	4.41	0.158	^1^(π_1_π_1_*)	37	8.13	T_4_	4.55	0.000	^3^(n_S_π_2_*)	49	T_4_	4.55	0.000	^3^(n_S_π_2_*)	49	S_3_^1^(π_2_π_1_*)	4.39	0.04		5.08
			^1^(π_S_π_1_*)	21																
			de ^1^(2π_S_π_1_*)	11																
T_4_	4.56		^3^(n_S_π_2_*)	52	3.04	S_3_	4.61	0.000	^1^(n_S_π_2_*)	49	S_3_	4.61	0.000	^1^(n_S_π_2_*)	49					
			de ^3^(π_S_π_1_*+ n_S_π_1_*)	17																
S_4_	4.90	0.128	^1^(π_S_π_2_*)	42	3.83	S_4_	4.82	0.008	^1^(π_1_π_1_*)	48	S_4_	4.93	0.018	^1^(π_1_π_1_*)	45	S_4_^1^(ππ*)			5.12	
			^1^(π_1_π_1_*)	18																
T_5_	4.93		^3^(π_1_π_2_*)	50	3.95															
S_5_	5.10	0.000	^1^(n_S_π_2_*)	57	3.14															
			de ^1^(π_S_π_1_*+ n_S_π_1_*)	13																
			de ^1^(π_1_π_1_*+ n_S_π_1_*)	11																
T_6_	5.19		^3^(π_S_π_2_*)	29	5.69															
			^3^(π_1_π_1_*)	17																
			^3^(π_S_π_1_*)	14																
			^3^(π_1_π_2_*)	10																
S_6_	6.05	0.000	^1^(n_O_π_1_*)	70	11.45															
T_7_	6.19		^3^(n_O_π_2_*)	63	5.37															
			^3^(n_O_π_3_*)	14																

As we can notice, the 4-TU brightest state is the S_2_ (^1^π_S_π_1_*) state, derived from the ground state by a single electron excitation from the π_S_ to the π_1_* orbital, with a smaller contribution from the π_1_ → π_1_* single excitation, which has also a contribution from the S atom (Figure 2) and also a small influence of double excitations from the π_S_ orbital. The S_1_ (^1^n_S_π_1_*) appears at 2.75 eV vertically above the ground state, being derived from it by a single excitation from the n_S_ lone pair orbital, mainly localized on the sulfur atom, to the π_1_* orbital; the associated oscillator strength is computed to be null. Below the S_1_ (^1^n_S_π_1_*) state, we found two lowest-lying triplet electronic states, T_1_^3^(π_S_π_1_*,π_1_π_1_*) and T_2_^3^(n_S_π_1_*). The former has an electronic wave function mainly dominated by the ^1^π_S_ → π_1_* single excitation and is localized at 2.59 eV vertically above the ground state, and the latter is mainly described by the n_S_ → π_1_* single excitation and appears at 2.62 eV above the ground state energy. It is worth noticing the presence of triplet states below the S_1_^1^(n_S_π_1_*) and S_2_^1^(π_S_π_1_*) singlet excited states, a favorable condition for triplet state population via intersystem crossing mechanisms.

Regarding TDDFT and TDA-computed data, at the TDDFT level, the S_2_^1^(π_S_π_1_*) is 0.22 eV higher in energy than that at the CASPT2 level. This state is vertically placed at 4.49 eV at the TDA level, namely, about 0.5 eV overestimated with respect to the CASPT2 value. Both DFT values are much higher than the experimental one, obtained in water and in acetonitrile (3.79 eV).^39,41^ This discrepancy on the S_2_^1^(π_S_π_1_*) state energy between DFT-based and multiconfigurational methods might be due to the multiconfigurational nature with certain contribution of double excitations, as identified in Table 1. Furthermore, the TDDFT and TDA methods were not able to reproduce the CASPT2 energetic order, in particular for the states situated above the S_2_^1^(π_S_π_1_*) state, where also some double excitations were found in the description of the electronic states. This suggests a limitation in the ability of the TDDFT and TDA methodologies mentioned here to provide an accurate description of the spectroscopic data of 4-TU.

The *E*_v_^abs^ computed herein are now compared with those obtained experimentally.^39^ The experimental absorption spectrum exhibits two bands, the most intense peaking at ∼327 nm (3.79 eV, ranging from 280 to 360 nm) and the weakest at 240 nm (5.12 eV). According to our results, the strongest absorption band can be associated with the electronic transitions to the S_2_^1^(π_S_π_1_*) and S_3_^1^(π_1_π_1_*) excited states. The electronic transition to the S_2_^1^(π_S_π_1_*) can be associated with the maximum of the strongest band at 3.79 eV, while the S_3_^1^(π_1_π_1_*) state contributes to the higher energy region. The weakest band can be assigned to the transition to the S_4_^1^(π_S_π_2_*) state, located at 4.90 eV (253 nm) above the ground state.

### Comparison of Geometries by Different Levels of Theory

**Figure 3:**
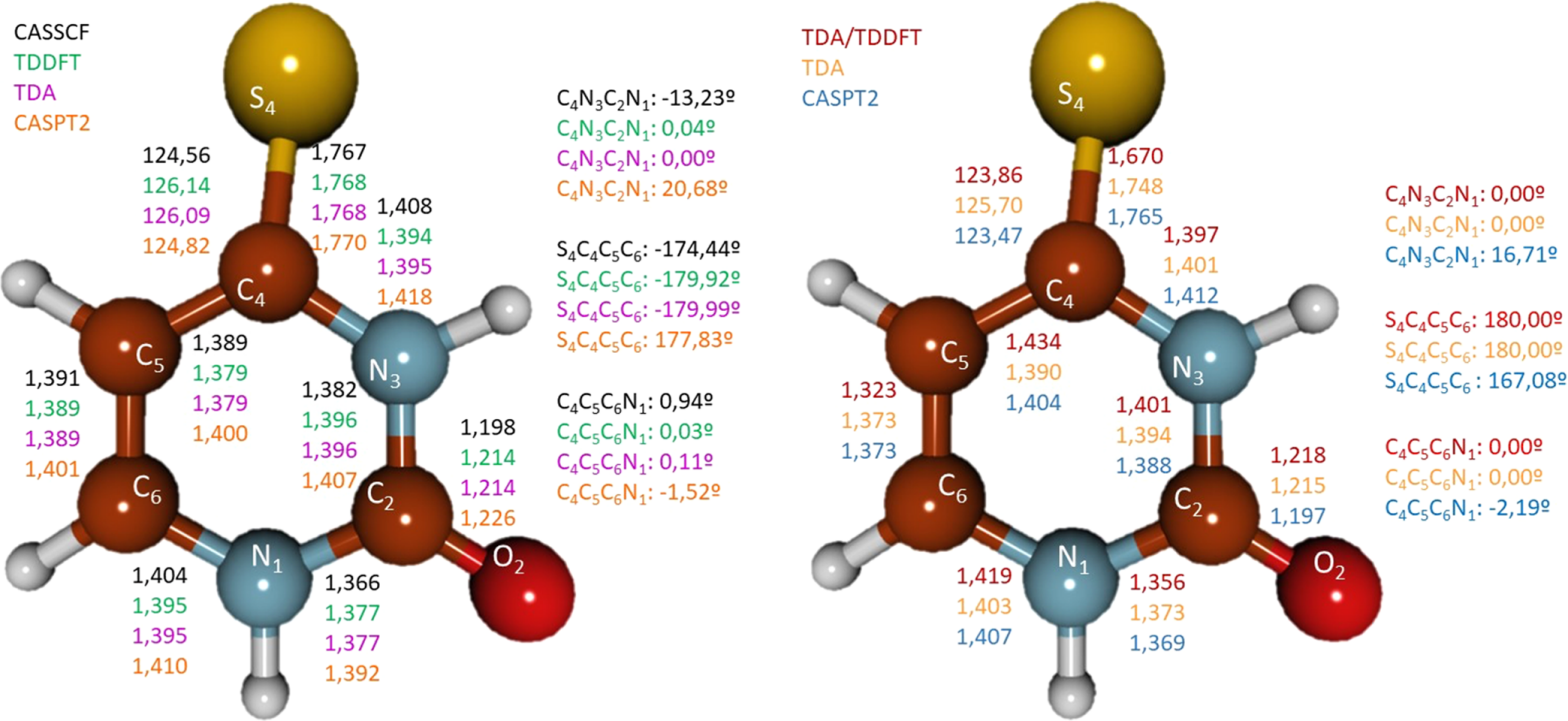
Most relevant geometrical parameters for 4-thiouracil S_1_T_1_ (^1^n_S_π_1_*/^3^π_S_π_1_*) singlet–triplet crossing (left) and T_2_T_1_ (^3^n_S_π_1_*/^3^π_S_π_1_*) conical intersection (right) optimized at different levels of theory. Bond lengths are expressed in Å and angles and dihedrals in degrees (deg).

The performance of different methods for geometry optimizations (minima and minimum energy crossing points) was also analyzed to compare their accuracy. Thus, GS (S_0_ minimum) geometry was optimized with CASSCF, CASPT2, and DFT (Figure S1). In the case of the relevant points along the 4-TU decay paths, in particular, S_1_T_1_ (^1^n_S_π_1_*/^3^π_S_π_1_*) STC (equivalent to the S_1_ minimum), T_2_T_1_ (^3^n_S_π_1_*/^3^π_S_π_1_*) CI, S_2_^1^(π_S_π_1_*) minimum, T_2_^3^(n_S_π_1_*) minimum, T_1_^3^(π_S_π_1_*) minimum, S_2_S_1_ (^1^π_S_π_1_*/^1^n_S_π_1_*) CI, S_1_S_0_ (^1^n_S_π_1_*/GS) CI, T_1_S_0_ (^3^π_S_π_1_*/GS) STC, and T_2_S_0_ (^3^n_S_π_1_*/GS) STC (Figures 3 and S2–S8) were optimized with some or all of the CASSCF, CASPT2, TDDFT, TDA, and SF-TDDFT methods.

For the GS, DFT agrees with the CASSCF and CASPT2, providing all of them nearly planar geometries (see Figure S1). For the excited-state points, the highest discrepancies appear for the S_1_T_1_ (^1^n_S_π_1_*/^3^π_S_π_1_*) STC, which, as mentioned above, corresponds to the S_1_^1^(n_S_π_1_*) minimum (see Figure 3) and the T_2_T_1_ (^3^n_S_π_1_*/^3^π_S_π_1_*) CI (see Figure 3), which is nearby the T_2_^3^n_S_π_1_* minimum (Figure S3). Here, the most controversial parameters are the S_4_–C_4_–C_5_–C_6_, C_4_–C_5_–C_6_–N_1_, and C_4_–N_3_–C_2_–N_1_ dihedral angles, responsible for the out-of-plane distortions of the molecule. The optimized geometries at the TDDFT and TDA levels are mostly planar, while both the CASSCF and CASPT2 give rise to distorted structures.

**Figure 4:**
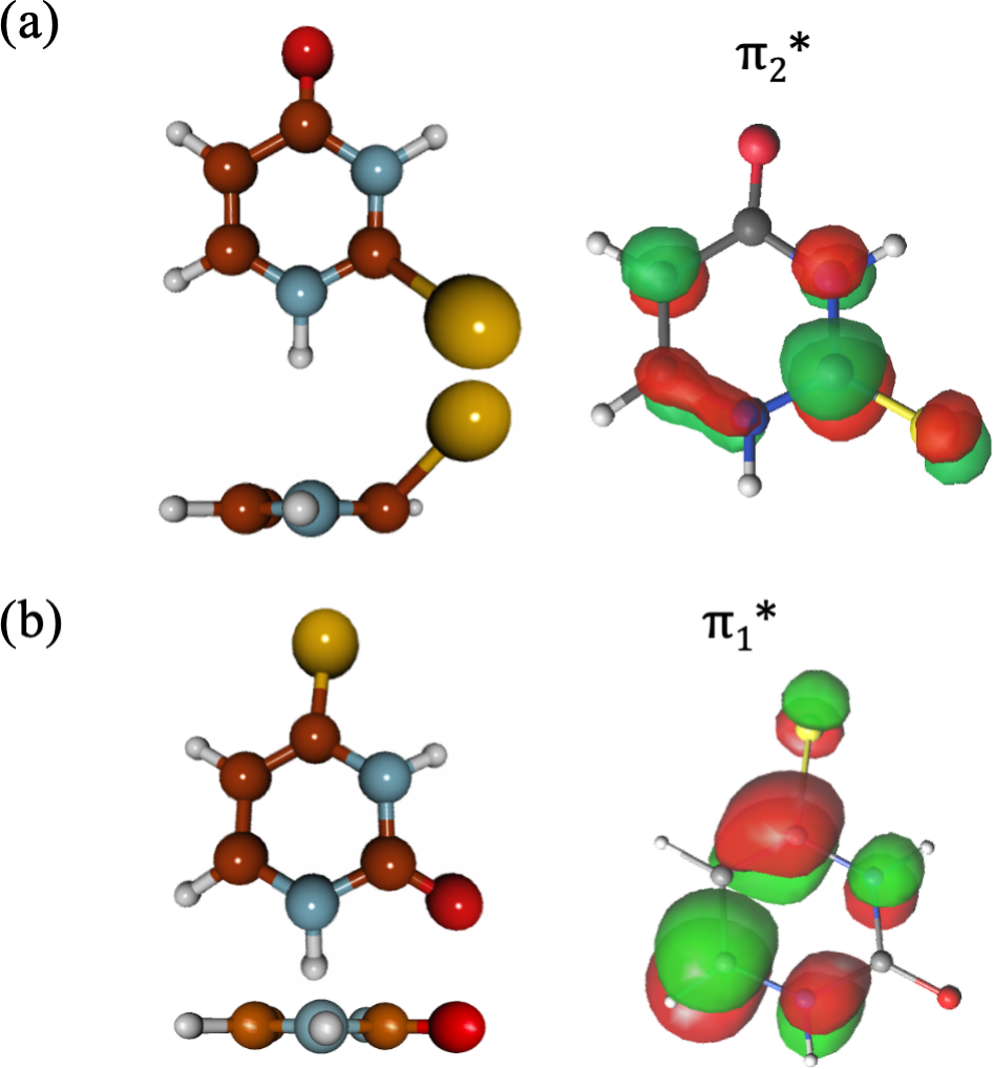
Relevant molecular orbitals for describing the bright states of (a) 2-thiouracil and (b) 4-thiouracil.

According to Mai et al.,^36^ 2-TU MS-CASPT2 optimized geometries are very distorted, mainly due to the C=S bond which lies out of the plane in some critical points. Due to the chemical resemblance between 2-TU and 4-TU, we would expect similar geometries for both systems. Nonetheless, deeper analyses have shown that their bright states are of different nature and, consequently, different geometries might be possible. For 2-TU, a relevant molecular orbital in the excited-state chemistry of this molecule is π_2_*, as labeled in ref (36), mainly localized on the C_2_ atom (see Figures 4 and S9 for the complete active space). Electron excitations from n_S_ or π_S_ orbitals to π_2_* increase the electronic density over the C_2_ atom, which becomes a carbanion with a pyramidalized structure, distorting the ring. On the other hand, the most relevant states of 4-TU involve an excitation to the π_1_* orbital, delocalized over the entire molecule (see Figure 4). In addition, there is a π bonding component on the C_4_ atom that extends to the adjacent carbon C_5_, making the pyramidalization of the C_4_ atom more difficult. This reasoning-based monoconfigurational description would prevent the out-of-plane distortion. The multiconfigurational description also indicates the lack of distortion, as described above. To verify whether the planar geometries obtained were a result of utilizing a small basis set (i.e., ANO-S-VDZP), optimizations of the S_1_, S_2_, and T_1_ minima were performed at the CASSCF(14,10)/ANO-L-VTZP level. In this case, the same planar structures were obtained as with the previous basis set. Additionally, a pyramidalized geometry was found for the minimum of the S_2_ excited state. This pyramidalized structure is located 0.63 eV higher in energy compared to the planar minimum. Consequently, we infer that it will not be relevant in our proposed scheme and that geometries obtained with ANO-S are trustable enough. The accuracy of the ANO-S basis set was demonstrated by Merchán et al. in related systems.^58^ The same tests were performed for the S_2_ and S_1_ minima of the 2-TU molecule obtaining, in both cases, both a planar and a pyramidalized structure. Energies can be found in Table S11. For the S_2_ equilibrium structure, both geometries are degenerated in energy, while for the S_1_ minima, the planar geometry is located 0.15 eV above the distorted one. These calculations agree with the vision described by Mai et al.,^36^ confirming that our level of theory is adequate for describing these systems.

Duan et al. used the TDDFT CAM-B3LYP/6-31G* approach to study the photochemistry and photophysics of 2-TU,^59^ obtaining similar geometries to those obtained by the MS-CASPT2 method. To analyze the performance of TDDFT in 4-TU, we also optimized the T_2_^3^(n_S_π_1_*) state using the CAM-B3LYP/6-31G* calculation level. The out-of-plane distortion obtained at the CASSCF and CASPT2 levels is not reproduced at the CAM-B3LYP level (Figure S3). This indicates that TDDFT and TDA methods, at least with the mentioned functional and basis set, are not accurate for the 4-TU photochemical pathways determination.

In contrast to TDDFT and TDA, SF-TDDFT provides a good agreement with CASSCF and CASPT2 in the T_1_S_0_ (^3^π_S_π_1_*/GS)_STC_ crossing structure computed at such a level (see Figure S7), similar to the observations found in the literature for 2-TU.^1,26^

Overall, we chose the CASSCF method, which is in good agreement with CASPT2 results for 4-TU, to obtain the geometries of the decay channels with some data computed with the SF-TDDFT method to provide comparisons with 2-TU from the literature using such a method.

### Photophysics of 4-Thiouracil

To understand the deactivation mechanisms of 4-TU excited states, we have performed MEP computations and CI and STC searches at the CASPT2//CASSCF level involving the S_0_, S_1_, S_2_, T_1_, T_2_, and T_3_ electronic states. CASSCF and CASPT2 energies of all states at the most relevant points of the photophysics of the system are shown in Tables S9 and S10.

**Figure 5:**
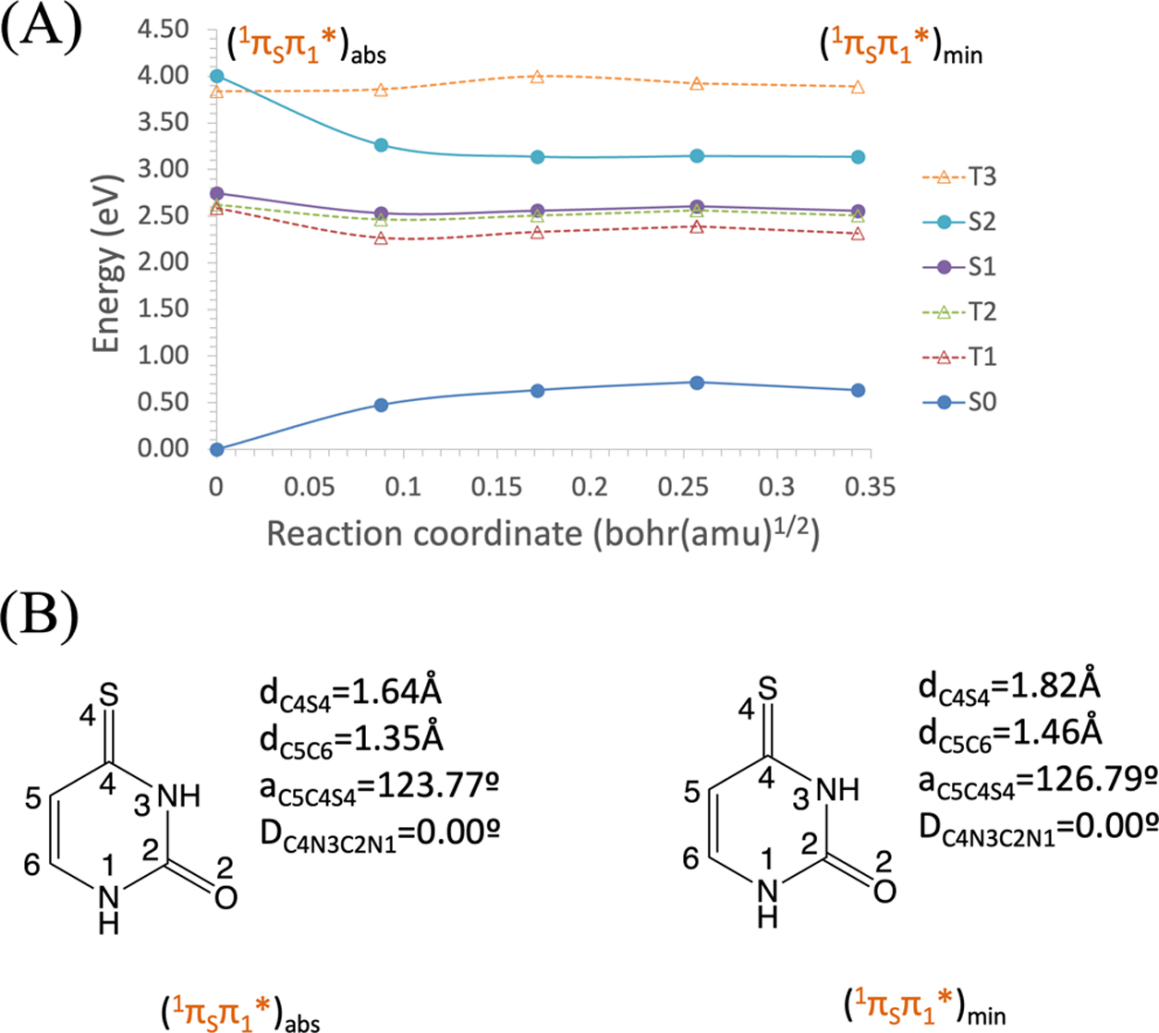
(A) Energies of the ground and lowest-lying singlet and triplet electronic excited states of 4-thiouracil along the minimum energy path (MEP) of the S_2_^1^(π_S_π_1_*) state from the S_0_ equilibrium structure toward the S_2_^1^(π_S_π_1_*) equilibrium geometry, computed at the CASPT2//CASSCF(14,10)/ANO-S-VDZP level of theory. (B) Main geometrical changes related with the MEP coordinate.

The initial step was to compute the MEP along the S_2_ bright excited-state potential energy hypersurface, starting from the FC region. The relative energies to the GS of the lowest singlet and triplet states along the MEP of the S_2_ state are shown in Figure 5A. In Figure 5B, the main geometrical changes that take place along the MEP coordinate are represented. As can be noticed, the S_2_ state evolves barrierless from the FC region to its minimum structure (last point of the MEP), which lies at 3.14 eV. When we remove the MEP constraint and optimize the geometry of this point, a nearby structure is found, slightly more stable (3.02 eV), which only differs in the N_3_C_3_S_4_ angle, which changes from 112.37 to 110.86°. It is important to note that this structure remains planar. It is also worth noticing the existence of an STC between the S_2_ and T_3_ states at the FC region with an SOC of <0.5 cm^–1^, very small to be responsible for a population transfer to T_3_ in this region. Therefore, this STC can be safely ignored as a viable triplet state population mechanism.

As the S_2_^1^(π_S_π_1_*) state does not cross other potential energy hypersurfaces along its MEP from the FC region, we continued searching for a possible MECP (CI and STC) in other regions. A multistate crossing region was found involving the S_1_^1^(n_S_π_1_*), S_2_^1^(π_S_π_1_*), T_1_^3^(π_S_π_1_*), and T_2_^3^(n_S_π_1_*) states. From there on, several possible paths are open for the decay. The CI between S_2_^1^(π_S_π_1_*) and S_1_^1^(n_S_π_1_*) is found at 3.37 eV. This crossing point is located ∼0.24 eV above the S_2_^1^(π_S_π_1_*) minimum and is similar to the CI reported for 2-TU in ref (43). The connection between the minimum and the CI is confirmed by an MEP computation on the S_2_^1^(π_S_π_1_*) state starting at the S_2_S_1_ (^1^π_S_π_1_*/^1^n_S_π_1_*) CI geometry (see Figure S10). It is worth mentioning that other S_2_S_1_ CIs were found at much higher energies, which makes them photochemically irrelevant. The computed geometries are displayed in Figure S5.

At the multistate crossing region, the STC between the S_2_^1^(π_S_π_1_*) and T_2_^3^(n_S_π_1_*) states is found at 3.41 eV, that is, 0.27 eV above the S_2_^1^(π_S_π_1_*) minimum. In this region, the SOC is computed to be 107.4 cm^–1^, a relatively large value, which can be attributed to the different nature of the states involved.

At the multistate crossing region, we also computed the SOC between other nearby singlet and triplet states. For the STC between the T_2_^3^(n_S_π_1_*) and S_1_^1^(n_S_π_1_*) states, the SOC is computed to be 7.3 cm^–1^, in agreement with ref (43). In the case of T_1_^3^(π_S_π_1_*) and S_1_^1^(n_S_π_1_*), the SOC is 169.3 cm^–1^. Finally, for S_2_^1^(π_S_π_1_*) and T_1_^3^(π_S_π_1_*), it is 6.0 cm^–1^. As can be seen, the lower or higher SOC follows El-Sayed’s rules.

From the multistate crossing region involving the S_1_^1^(n_S_π_1_*), S_2_^1^(π_S_π_1_*), T_1_^3^(π_S_π_1_*), and T_2_^3^(n_S_π_1_*) electronic states and considering the computed SOC, three plausible deactivation pathways are foreseen: (i) along the S_1_ state via the S_2_S_1_ (^1^π_S_π_1_*/^1^n_S_π_1_*) CI, (ii) along the T_2_^3^(n_S_π_1_*) state via the S_2_T_2_ (^1^π_S_π_1_*/^3^n_S_π_1_*) STC, and (iii) along the T_1_^3^(π_S_π_1_*) state via, first, the S_2_S_1_ (^1^π_S_π_1_*/^1^n_S_π_1_*) CI crossing and, next, the S_1_T_1_ (^1^n_S_π_1_*/^3^π_S_π_1_*) STC (note that these CIs and STCs appear to be near-degenerate). Thus, we decided to calculate the corresponding MEPs along the S_1_^1^(n_S_π_1_*), T_1_^3^(π_S_π_1_*), and T_2_^3^(n_S_π_1_*) potential energy hypersurfaces.

**Figure 6:**
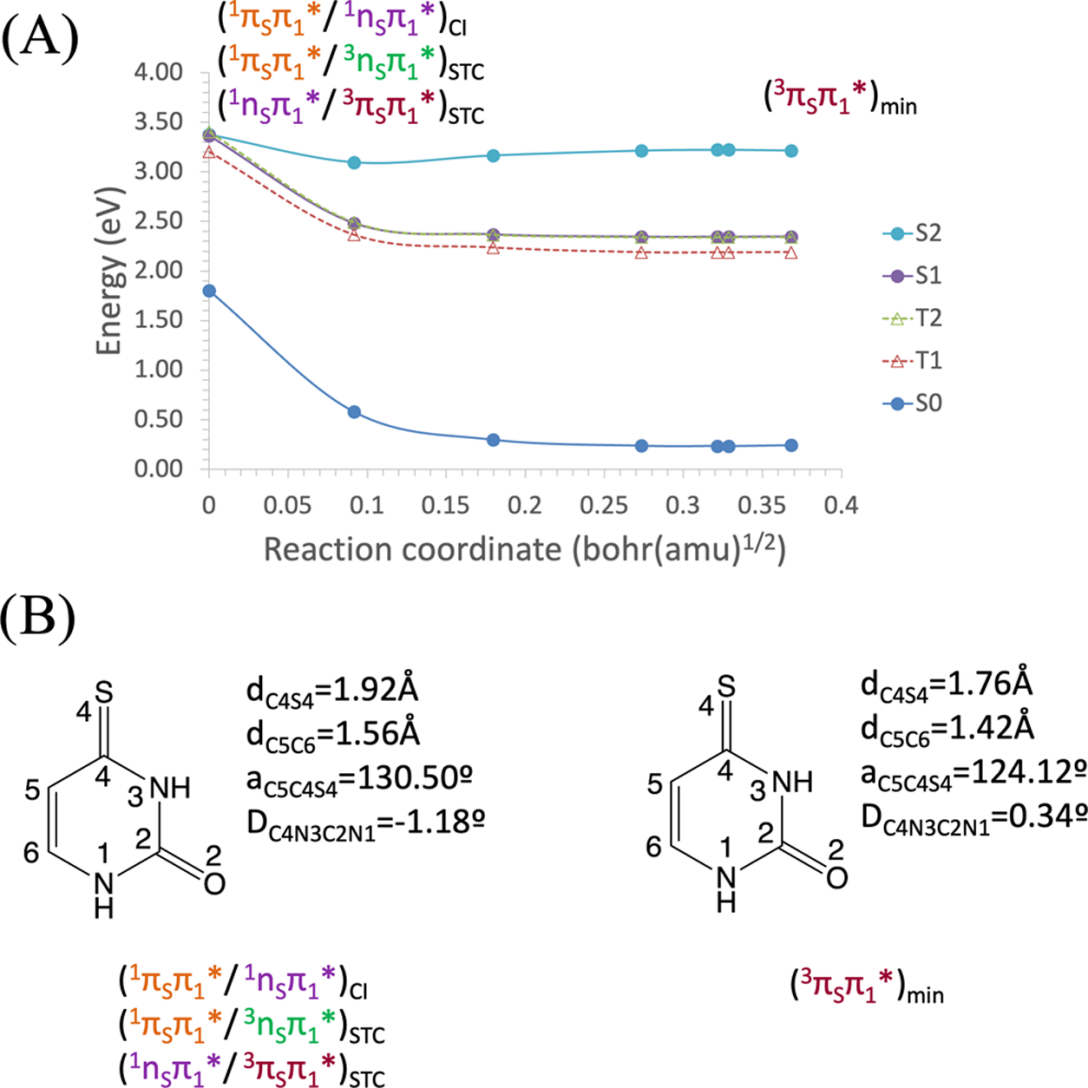
(A) Energies of the ground and lowest-lying singlet and triplet electronic excited states of 4-thiouracil along the minimum energy path (MEP) of the T_1_^3^(π_S_π_1_*) state from the S_2_S_1_ (^1^π_S_π_1_*/^1^n_S_π_1_*) conical intersection toward the T_1_^3^(π_S_π_1_*) equilibrium geometry, computed at the CASPT2//CASSCF(14,10)/ANO-S-VDZP level of theory. (B) Main geometrical changes related with the MEP coordinate.

**Figure 7:**
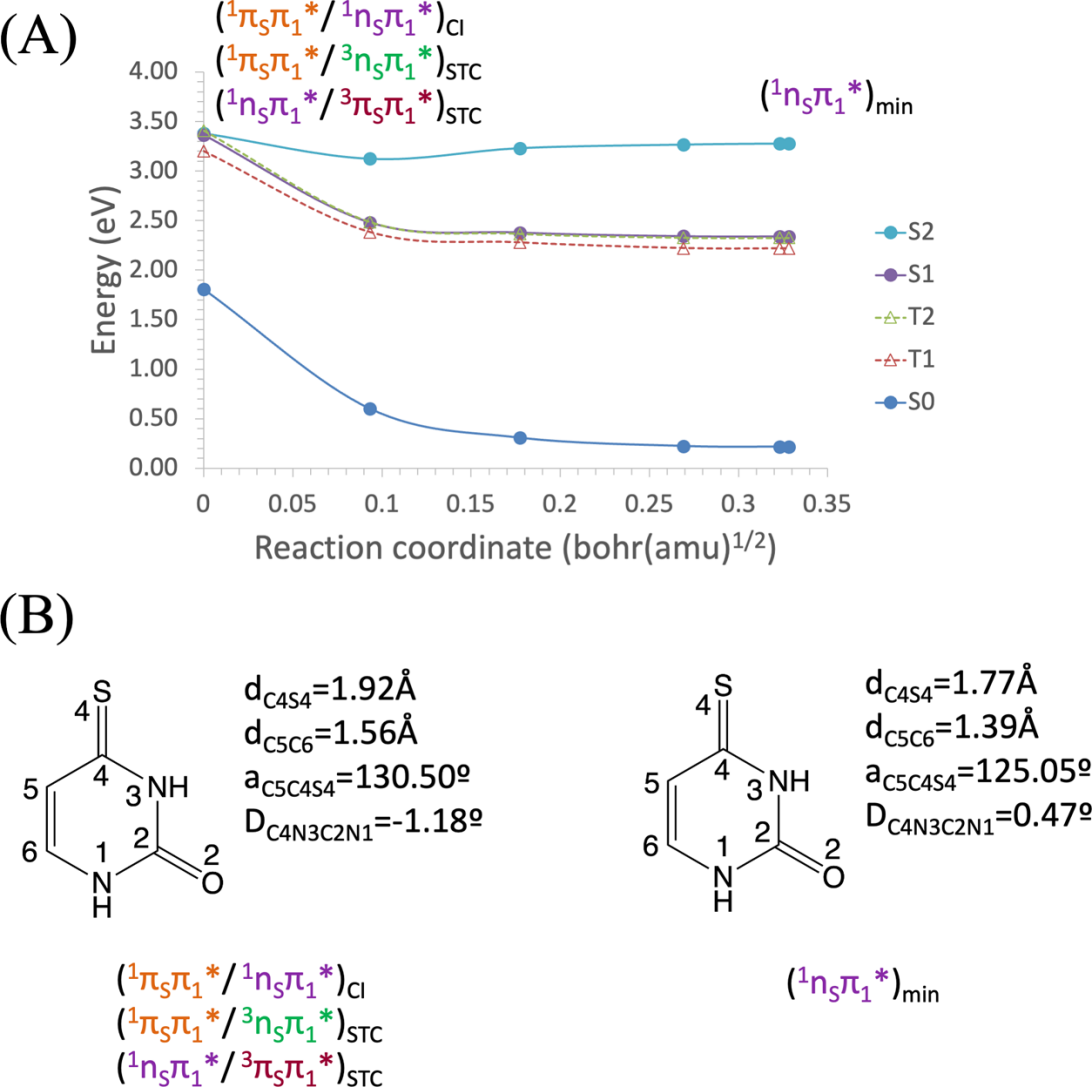
(A) Energies of the ground and lowest-lying singlet and triplet electronic excited states of 4-thiouracil along the minimum energy path (MEP) of the S_1_^1^(n_S_π_1_*) state from the S_2_S_1_ (^1^π_S_π_1_*/^1^n_S_π_1_*) conical intersection toward the S_1_^1^(n_S_π_1_*) equilibrium geometry, computed at the CASPT2//CASSCF(14,10)/ANO-S-VDZP level of theory. (B) Main geometrical changes related with the MEP coordinate.

**Figure 8:**
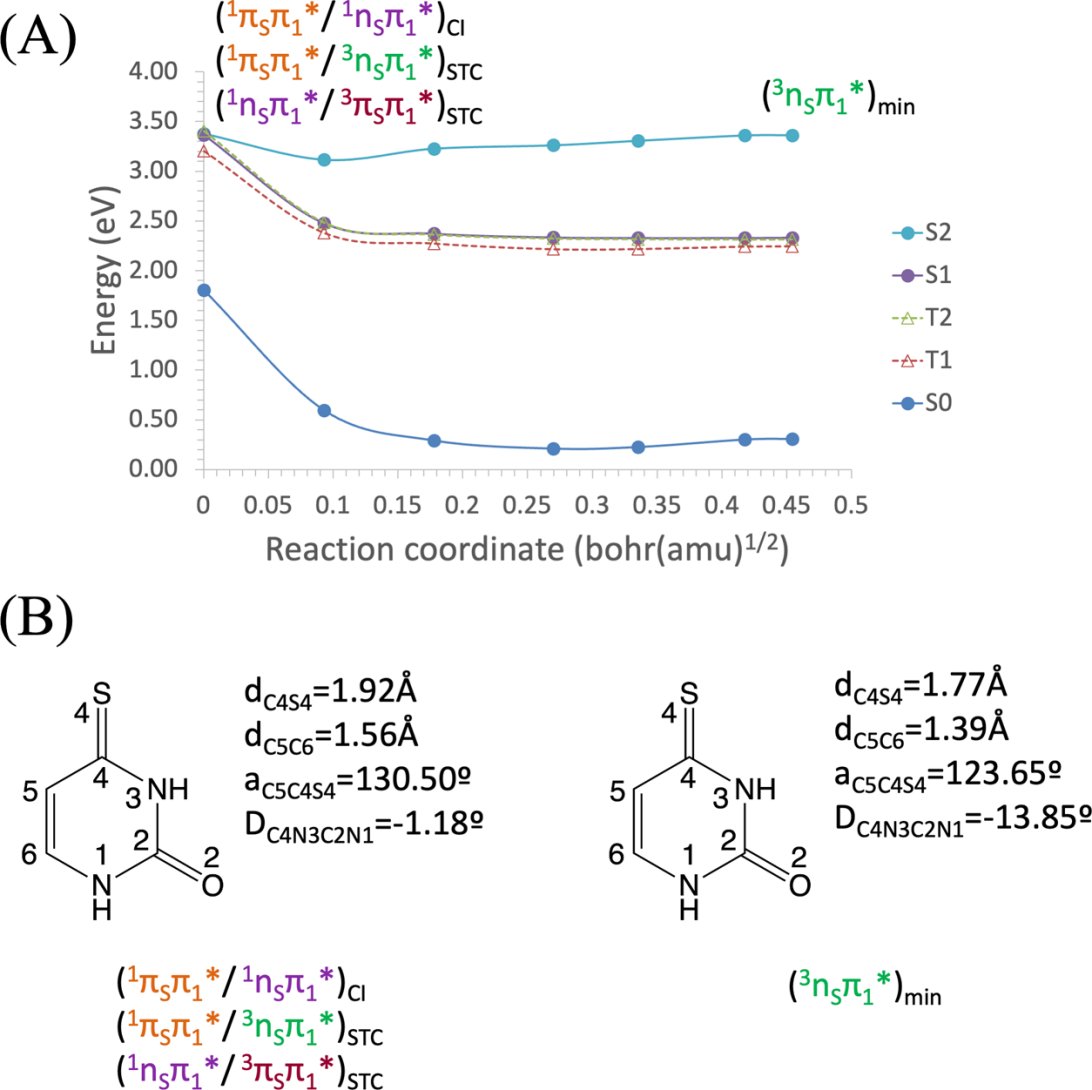
(A) Energies of the ground and lowest-lying singlet and triplet electronic excited states of 4-thiouracil along the minimum energy path (MEP) of the T_2_^3^(n_S_π_1_*) state from the S_2_T_2_ (^1^π_S_π_1_*/^3^n_S_π_1_*) singlet–triplet crossing toward the T_2_ (^3^n_S_π_1_*) minimum, computed at the CASPT2//CASSCF(14,10)/ANO-S-VDZP level of theory. (B) Main geometrical changes related with the MEP coordinate.

The T_1_^3^(π_S_π_1_*) MEP from the S_1_T_1_ (^1^n_S_π_1_*/^3^π_S_π_1_*)_STC_ ends in the minimum of that state at 2.19 eV, as shown in Figure 6. The S_1_^1^(n_S_π_1_*) MEP from the S_2_S_1_ (^1^π_S_π_1_*/^1^n_S_π_1_*)_CI_ (Figure 7) and the T_2_^3^(n_S_π_1_*) MEP from the S_2_T_2_ (^1^π_S_π_1_*/^3^n_S_π_1_*)_STC_ (Figure 8) show an evolution in parallel, as expected for nπ* states of carbonyls in π-conjugated molecules. The S_1_^1^(n_S_π_1_*) MEP ends barrierless at the equilibrium geometry of the S_1_^1^(n_S_π_1_*) state at 2.33 eV, which coincides with the S_1_T_1_ (^1^n_S_π_1_*/^3^π_S_π_1_*)_STC_. Meanwhile, the T_2_^3^(n_S_π_1_*) MEP also ends barrierless at its minimum, which is nearby the T_2_T_1_ (^1^π_S_π_1_*/^1^n_S_π_1_*)_CI_ at 2.31 eV. For each of these MEPs, geometry optimizations without the MEP constraint were performed, affirming that the final point in each case corresponds to the minimum of the respective state. As expected from the fact that the S_1_^1^(n_S_π_1_*) and T_1_^3^(π_S_π_1_*) states are of different nature, the computed SOC between them in the STC region is large (155.0 cm^–1^), in agreement with Borrego-Varillas et al.^43^

**Figure 9:**
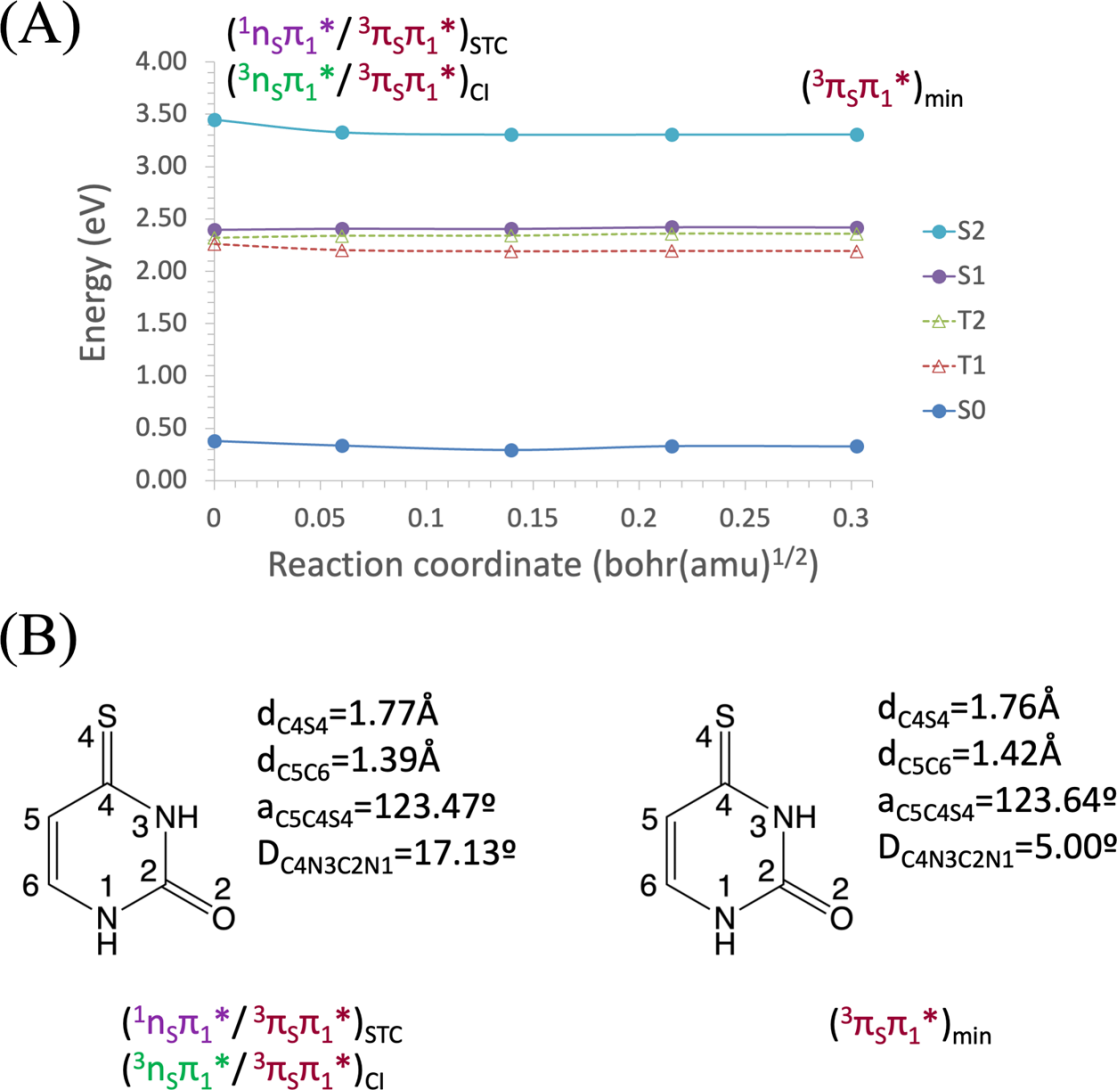
(A) Energies of the ground and lowest-lying singlet and triplet electronic excited states of 4-thiouracil along the minimum energy path (MEP) of the T_1_^3^(π_S_π_1_*) state from S_1_T_1_ (^1^n_S_π_1_*/^3^π_S_π_1_*) toward the T_1_^3^(π_S_π_1_*) equilibrium structure, computed at the CASPT2//CASSCF(14,10)/ANO-S-VDZP level of theory. (B) Main geometrical changes related with the MEP coordinate.

Finally, from the STC between the S_1_^1^(n_S_π_1_*) and T_1_^3^(π_S_π_1_*) states, the lowest-lying triplet state decays to its minimum, placed at 2.19 eV (see Figure 9). From this point, it can either transfer its energy to the triplet oxygen or deactivate itself to the GS through a radiative or a nonradiative process. To characterize the latter, we computed the T_1_S_0_ (^3^π_S_π_1_*/GS)_STC_, which was found at 3.17 eV. The MEP from the T_1_S_0_ (^3^π_S_π_1_*/GS)_STC_ along the T_1_^3^(π_S_π_1_*) potential energy surface connects the STC with the T_1_^3^(π_S_π_1_*) minimum, as shown in Figure S11. The relative energy from the T_1_ minimum to reach the T_1_S_0_ (^3^π_S_π_1_*/GS)_STC_ and therefore the point for nonradiative decay to the GS is ∼0.98 eV, which is relatively high. Furthermore, the SOC at this STC between T_1_ and S_0_ has a relatively low value of 5 cm^–1^. Both features make this process unfavorable.

The crossing between the S_0_ and S_1_^1^(n_S_π_1_*) electronic states was also computed for 4-TU, displaying energy barriers of about 1.15 eV from the S_1_^1^(n_S_π_1_*) minimum. Such a barrier is too high to play a relevant role in the deactivation mechanism of the molecule to the GS.

The most relevant geometry changes along the deactivation paths involve the S_4_–C_4_ and C_5_–C_6_ bonds, the S_4_–C_4_–C_5_ angle, and dihedral angles related to distortions from the molecular plane, namely, S_4_–C_4_–C_5_–C_6_, C_4_–C_5_–C_6_–N_1_, and C_4_–N_3_–C_2_–N_1_ (see Figures 3 and S1–S8). Along the S_2_^1^(π_S_π_1_*) state evolution from the FC toward the multistate crossing region, the above-mentioned chemical bonds elongate and the angles increase, while the molecule remains mostly planar. Then, from the CI between the S_2_^1^(π_S_π_1_*) and S_1_^1^(n_S_π_1_*) electronic states, the S_1_^1^(n_S_π_1_*) MEP evolves similarly toward the STC with T_1_^3^(π_S_π_1_*), shortening the bonds and decreasing the angles, but in this case, the molecule becomes slightly more distorted. The same happens from the STC between S_2_^1^(π_S_π_1_*) and T_2_^3^(n_S_π_1_*) on the T_2_^3^(n_S_π_1_*) MEP toward the CI with T_1_^3^(π_S_π_1_*). Finally, from the end of such MEPs on the T_1_^3^(π_S_π_1_*) potential energy hypersurface toward its minimum, the chemical bonds and angles remain almost invariant, but the molecule returns to a flatter configuration.

**Figure 10:**
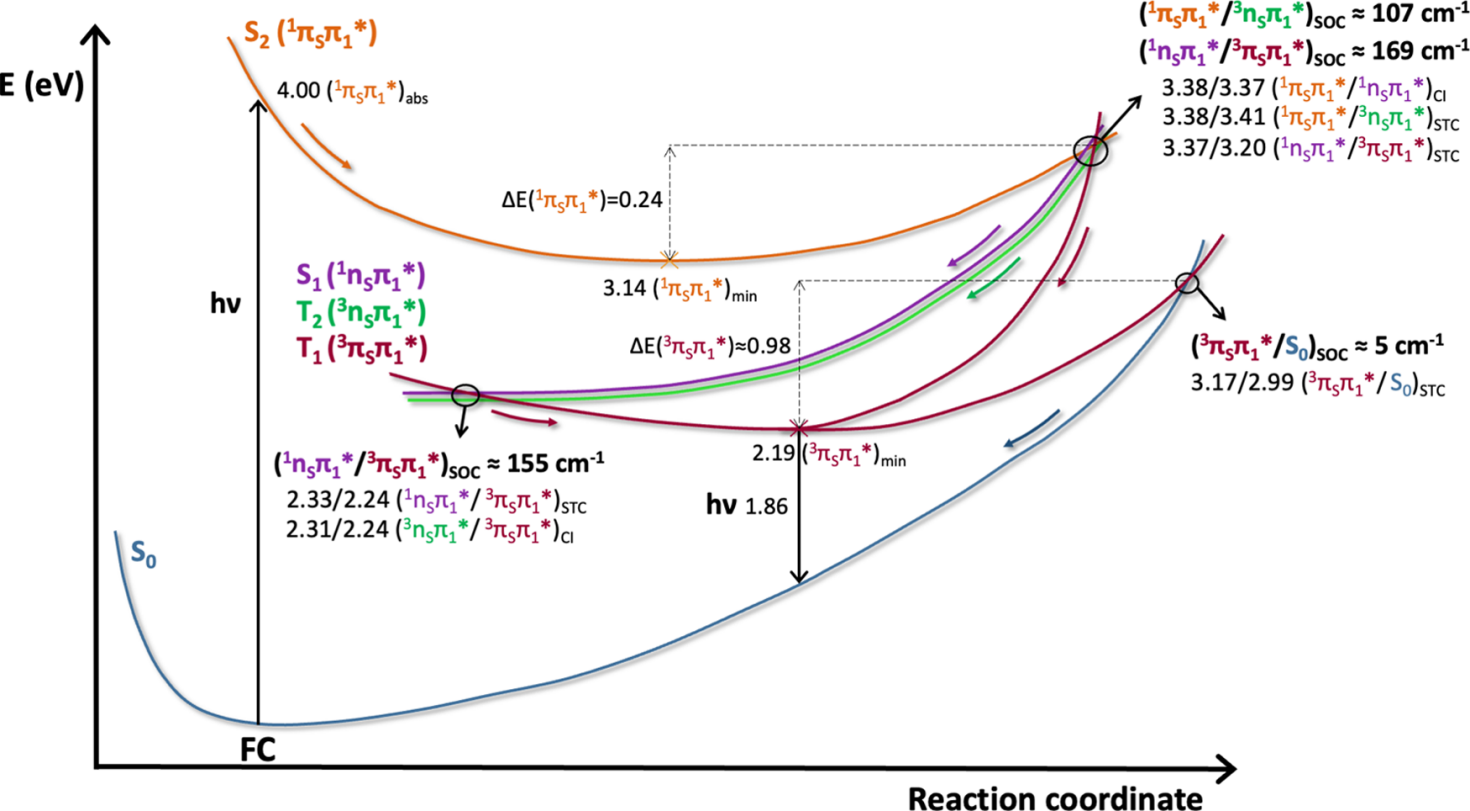
Schematic representation of the deactivation pathways for photoexcited 4-thiouracil based on the energy values obtained at the SA(7)-CASSCF(14,10)//CASPT2/ANO-S-VDZP level of theory. Main energy values (eV) of the vertical absorption, excited-state minima and minimum energy crossing points, energy barriers and spin–orbit couplings (cm^–1^) are also indicated in the figure.

Figure 10 shows an illustrative scheme of the decay paths derived from these SS-CASPT2 computations. The same scheme has been also represented with MS-CASPT2 energy values, see Figure S12 in the SI, with no significant differences on the main features of the photophysics mechanism, which reinforces the accuracy of the description provided. Three deactivation pathways can be proposed for photoexcited 4-TU. The first one will be S_2,FC_ (4.0 eV) → S_2,min_ (3.1 eV) → S_1,(S2S1)CI_ (3.4 eV) → S_1,min_ (2.3 eV) → T_1,(S1T1)STC_ (2.3 eV) → T_1,min_ (2.2 eV), with an SOC of 155 cm^–1^ between the S_1_^1^(n_S_π_1_*) and T_1_^3^(π_S_π_1_*) states. The second path will be S_2,FC_ (4.0 eV) → S_2,min_ (3.1 eV) → S_1,(S2S1)CI_ (3.4 eV) → T_1,(S1T1)STC_ (3.4 eV) → T_1,min_ (2.2 eV), with an SOC of 169 cm^–1^ between the S_1_^1^(n_S_π_1_*) and T_1_^3^(π_S_π_1_*) states. The last one will be S_2,FC_ (4.0 eV) → S_2,min_ (3.1 eV) → T_2,(S2T2)STC_ (3.4 eV) → T_1,(T2T1)CI_ (2.3 eV) → T_1,min_ (2.2 eV), with an SOC of 107 cm^–1^ between the S_2_^1^(π_S_π_1_*) and T_2_^3^(n_S_π_1_*) states. The large SOC observed along the second path points out that it would be the most likely to happen. On the other hand, in the first path, the spin inversion occurs in the minimum of the S_1_^1^(n_S_π_1_*) state, where the molecule will be trapped, increasing the probability for ISC even with lower SOC values. Thus, both regions shall allow for a significant ISC mechanism. However, as they correspond to the same mechanism, we will refer to them as S_2_^1^(π_S_π_1_*) → S_1_^1^(n_S_π_1_*) → T_1_^3^(π_S_π_1_*) and the last one as S_2_^1^(π_S_π_1_*) → T_2_^3^(n_S_π_1_*) → T_1_^3^(π_S_π_1_*) paths. Therefore, we will have, in general, two deactivation pathways, with the first being the most probable.

### Comparison with 2-Thiouracil

An analogous scheme to the one presented in Figure 10 has been created for 2-TU (Figure S13), in order to facilitate the comparison with 4-TU. Data were obtained from refs^36 and60^ computed at the SA(4/3)-CASSCF(12,9) + MS(3/3)-CASPT2/cc-pVDZ level of theory. In 2-TU, the most probable decay path is S_2_^1^(π_S_π*) → S_1_^1^(n_S_π*) → T_2_/T_1_^3^(π_S_π*), with an SOC between the S_1_^1^(n_S_π_1_*) and T_2_^3^(π_S_π_1_*) states about 150 cm^–1^.^36^ The energetic barriers between the S_2_^1^(π_S_π_1_*) minimum and the S_2_S_1_ (^1^π_S_π_1_*/^1^n_S_π_1_*) CI of both the molecules are relatively small (0.24 eV in 4-TU, as reported here, and less than 0.1 eV in 2-TU).^36^ In addition, the SOCs are larger for 4-TU. Therefore, based on the SOC values, we can say that 4-TU decays more efficiently to the lowest-lying triplet state than 2-TU, whereas if we consider the energetic barriers between the S_2_^1^(π_S_π_1_*) minimum and the S_2_S_1_ (^1^π_S_π_1_*/^1^n_S_π_1_*) CI, 2-TU seems to decay more favorably. The first case agrees with the experimental measurements for the triplet quantum yield, which is higher for 4-TU than for 2-TU (Φ_ISC_ = 0.90 ± 0.15^33,39,40^ and 0.75 ± 0.20,^33^ respectively). Hence, it seems that for such small energy barriers, the SOC is the dominant parameter. The triplet quantum yield is linked with the singlet oxygen formation yield, which is also very high for 4-TU (Φ_Δ_ = 0.49 ± 0.02).^33,41^ Moreover, this higher singlet oxygen formation is coherent with the fact that the phosphorescence yield measured in the presence of oxygen is lower for 4-TU (Φ_Ph_ = 0.15),^42^ whereas it is much larger in the case of 2-TU (Φ_Ph_ = 0.65–0.70).^30,31^ In this context, the distinct radiative rates (*k*_Ph_) from the T_1_ minimum to the GS are also contributing to the different phosphorescence experimental yields. According to our CASPT2 computations, the *k*_Ph_ is 3.3 s^–1^ for 4-TU, and for 2-TU, the *k*_Ph_ values are 2403.1 and 35.2 s^–1^ for the two T_1_ minima reported in the literature (π_S_π_2_* and π_5_π_6_*, respectively).^36^

As mentioned in the previous section, the more accessible nonradiative path, which reduces the lifetime of T_1_, corresponds to the T_1_S_0_ (^3^π_S_π_1_*/GS)_STC_ found at 3.12 eV at the CASPT2 level. As in the literature there are available data for 2-TU at the SF-TDDFT level, for the sake of comparability, we also used this method for 4-TU. According to our results, there is only a T_1_^3^(π_S_π_1_*) minimum in 4-TU. Then, only a crossing of this state with the GS was obtained, located at 0.63 eV over the minimum at the SF-TDDFT level (about 1.00 eV at the CASPT2 level). The SOC between both states was computed to be 15.9 cm^–1^. In the case of 2-TU, two T_1_ minima (π_S_π_2_* and π_S_π_6_*) were found in the literature,^36,60^ very close energetically. Therefore, we searched for two crossings with the GS. The T_1_S_0_ (^3^π_S_π_2_*/GS)_STC_ and T_1_S_0_ (^3^π_S_π_6_*/GS)_STC_ are located at 0.29 and 0.25 eV, respectively, over their minima. The calculated SOC values for these crossings amount to 150.0 and 4.4 cm^–1^, respectively. These findings show that the T_1_S_0_ (^3^π_S_π_2_*/GS)_STC_ crossing is less accessible in 4-TU than in 2-TU. These theoretical results, together with the higher *k*_Ph_ values for triplet emission of 2-TU with respect to that of 4-TU mentioned above, allow us to interpret the longer triplet lifetime in 4-TU (τ = 0.23 ± 0.02 μs)^39^ than in 2-TU (τ = 0.07 ± 0.02 μs).^32^

## Conclusions

The relaxation mechanisms of 4-TU, a prominent photosensitizer for photodynamic therapy, were studied with CASPT2//CASSCF and DFT methodologies, and the photophysical properties were compared with those of 2-TU. We found that the former molecule absorbs light populating mainly the S_2_^1^(π_S_π_1_*) state, vertically located at 4.00 eV, and the most probable evolution implies the nonradiative deactivation toward the equilibrium structure of the lowest-lying triplet state, T_1_^3^(π_S_π_1_*), placed at an adiabatic energy of 2.19 eV. From the S_2_^1^(π_S_π_1_*) state, we devised two possible deactivation pathways to populate the mentioned lowest triplet state. A possible path is S_2_^1^(π_S_π_1_*) → S_1_^1^(n_S_π_1_*) → T_1_^3^(π_S_π_1_*), with an SOC in the range of 155.0 → 169.3 cm^–1^ between the S_1_^1^(n_S_π_1_*) and T_1_^3^(π_S_π_1_*) states. Another route corresponds to S_2_^1^(π_S_π_1_*) → T_2_^3^(n_S_π_1_*) → T_1_^3^(π_S_π_1_*), with an SOC of 107.4 cm^–1^ between the S_2_^1^(n_S_π_1_*) and T_2_^3^(n_S_π_1_*) states. The energy barriers along the paths are very low in both cases and therefore the SOC values will determine the ISC efficiencies and, consequently, the most probable deactivation path. Therefore, 4-TU will most probably decay through the path via S_1_^1^(n_S_π_1_*) as an intermediate state.

A difference between the lowest-lying triplet state in both systems was found. In the case of 4-TU, there is only a minimum in the T_1_^3^(π_S_π_1_*) potential energy hypersurface, while in 2-TU, it shows two minima of different nature (^3^(π_S_π_2_*) and ^3^(π_S_π_6_*)). In the 2-TU molecule, the T_1_S_0_ crossings are, in general, more accessible and have higher SOCs than in 4-TU. Therefore, the deactivation to the GS will be more favorable for 2-TU.

These mentioned findings were already obtained by Borrego-Varillas et al.^43^ modeling the excited-state dynamics of 4-TU in aqueous solution. Herein, differences in experimental data between 4-TU and 2-TU were further rationalized. Thus, the longer triplet lifetimes and lower phosphorescence quantum yields for the former can be explained due to the less energetically accessible T_1_S_0_ crossing, lower SOCs at such crossing, and a lower radiative rate at the T_1_ minimum. The larger ISC quantum yield of 4-TU with respect to that of 2-TU can be associated with the fact that the SOCs along the decay path toward the T_1_ equilibrium geometry are higher for 4-TU. The high ISC quantum yield of 4-TU is also linked to a high singlet oxygen formation and a lower phosphorescence yield measured under aerobic conditions.

Thus, with the present study, we reinforce the conclusion that 4-TU is a better photosensitizer compared to 2-TU and is deemed a more suitable candidate for photodynamic therapy.

## Supporting Information Available

Geometrical parameters of interest for 4-TU; CASSCF active space for the 2-TU system; complementary MEPs for the 4-TU deactivation mechanism with their respective geometrical changes; schematic representation of the deactivation pathway of 4-TU with MS-CASPT2 energies; schematic representation of the deactivation pathway of 2-TU; ANO-S and ANO-L basis set comparison at the Franck–Condon region and MECPs for energies and spin–orbit couplings; SS and MS-CASPT2 methods comparison at equilibrium structures and MECPs for energies and oscillator strengths; reference weights for SS-CASPT2 calculations; CASSCF and CASPT2 energies at relevant points of the 4-TU molecule; Cartesian coordinates (PDF)
