# Supplementary material for: Quantum-Chemistry Study of the Photophysical Properties of 4-Thiouracil and Comparisons with 2-Thiouracil
Source: J Phys Chem A. 2024 Mar 20;128(12):2273–85. doi: 10.1021/acs.jpca.3c06310 (PMC10982997; doi:10.1021/acs.jpca.3c06310)
Supplement: Supplementary file 1 — jp3c06310_si_001.pdf [file jp3c06310_si_001.pdf]

Supporting Information:

Quantum-Chemistry Study of the Photophysical Properties of 4-Thiouracil and Comparisons with 2-Thiouracil

Miriam Navarrete-Miguel,[a] Angelo Giussani,[a] Mercedes Rubio,[b] Martial Boggio-Pasqua,[c] Antonio Carlos Borin,[d] and Daniel Roca-Sanjuán,[a]*

[a] Instituto de Ciencia Molecular, Universitat de València, P.O. Box 22085, ES-46071 Valencia, Spain.

[b] Departament de Química Física, Universitat de València, 46100 Burjassot, Spain

[c] Laboratoire de Chimie et Physique Quantiques, IRSAMC, CNRS et Université Toulouse 3, 118 route de Narbonne, 31062 Toulouse, France

[d] Department of Fundamental Chemistry, Institute of Chemistry, University of São Paulo, Av. Prof. Lineu Prestes, 748, São Paulo, SP, CEP 05508-000, Brazil

Supporting information

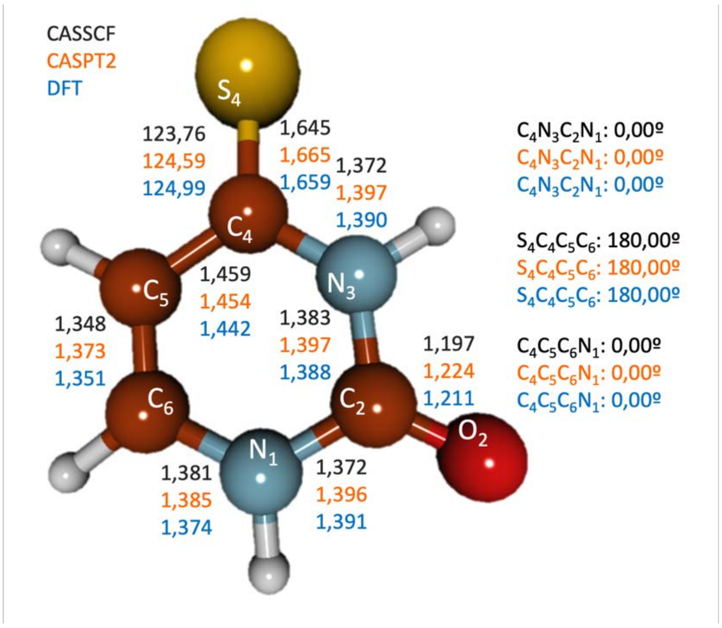

Figure S1. Most relevant geometrical parameters for the ground-state (S_0) equilibrium structure of 4TU optimized at different levels of theory. Bond lengths are expressed in Å and angles and dihedrals in degrees (º).

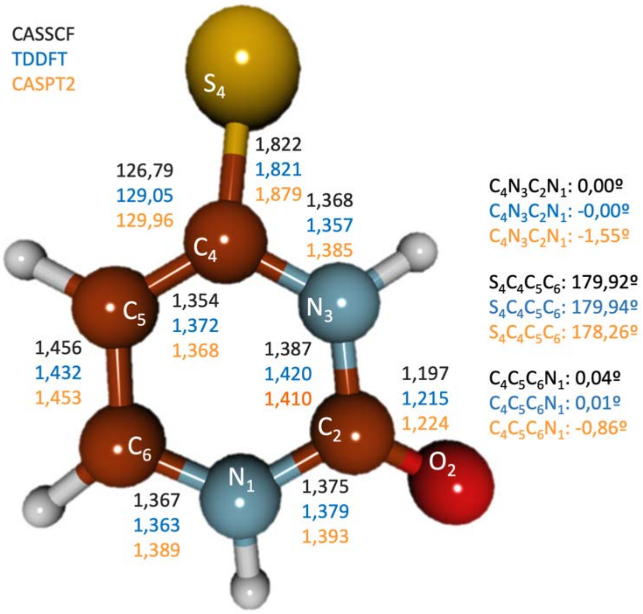

Figure S2. Most relevant geometrical parameters for the S_2 $^1(\pi_S\pi_1^*)$ excited state equilibrium structure of 4TU optimized at different levels of theory. Bond lengths are expressed in Å and angles and dihedrals in degrees (º).

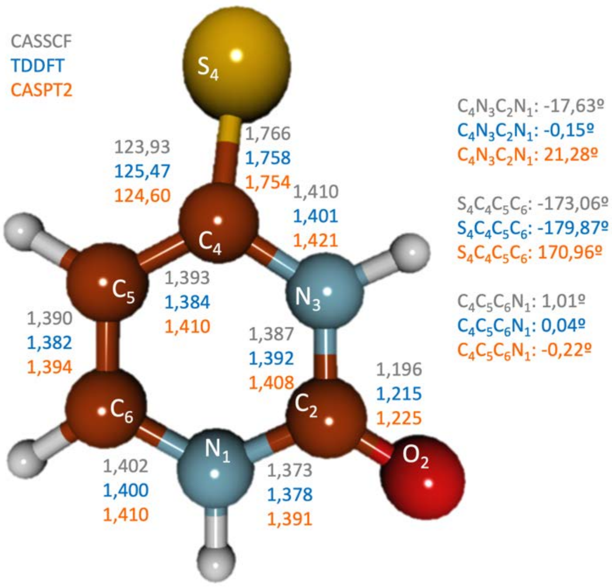

Figure S3. Most relevant geometrical parameters for the T_2 ($^3n_S\pi_1^*$) excited state equilibrium structure of 4TU optimized at different levels of theory. Bond lengths are expressed in Å and angles and dihedrals in degrees (º).

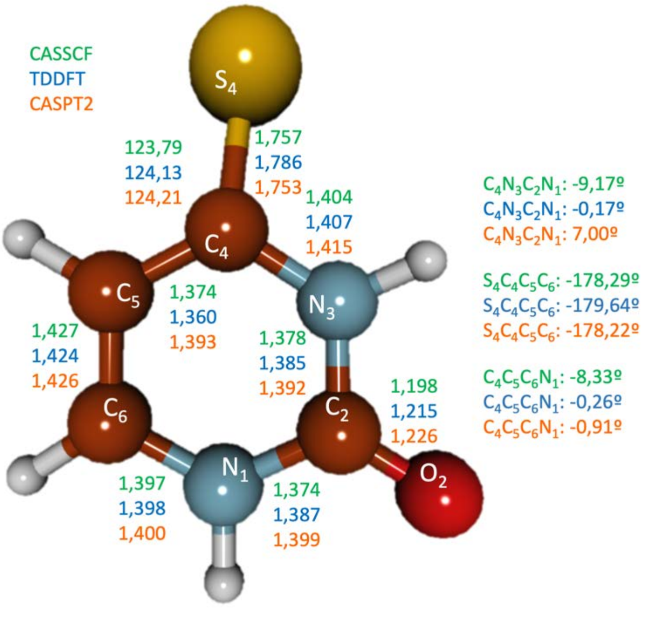

Figure S4. Most relevant geometrical parameters for the T_1 $^3(\pi_S\pi_1^*)$ equilibrium structure of 4TU optimized at different levels of theory. 4-Thiouracil. Bond lengths are expressed in Å and angles and dihedrals in degrees (º).

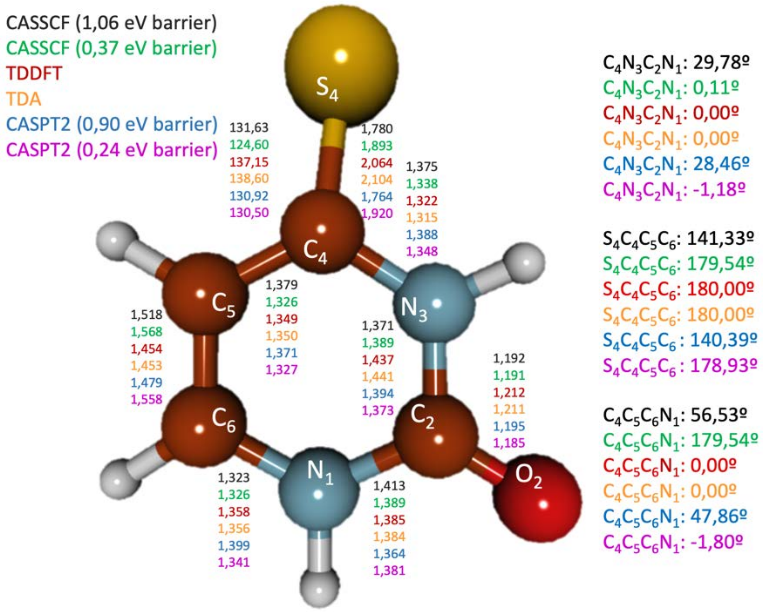

Figure S5. Most relevant geometrical parameters for the S_2S_1 ($^1\pi_S\pi_1$*/$^1n_S\pi_1$*) conical intersection structure of 4TU optimized at different levels of theory. Bond lengths are expressed in Å and angles and dihedrals in degrees (º).

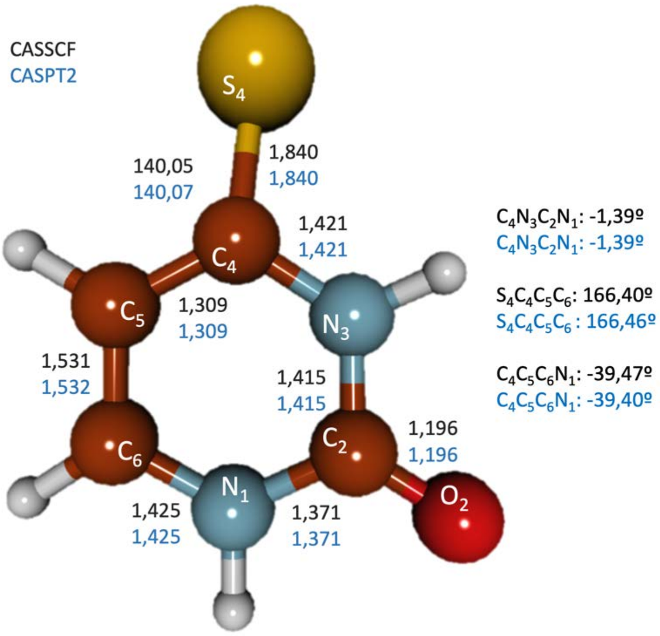

Figure S6. Most relevant geometrical parameters for the S_1S_0 ($^1n_S\pi_1$*/GS) conical intersection structure of 4TU optimized at different levels of theory. Bond lengths are expressed in Å and angles and dihedrals in degrees (º).

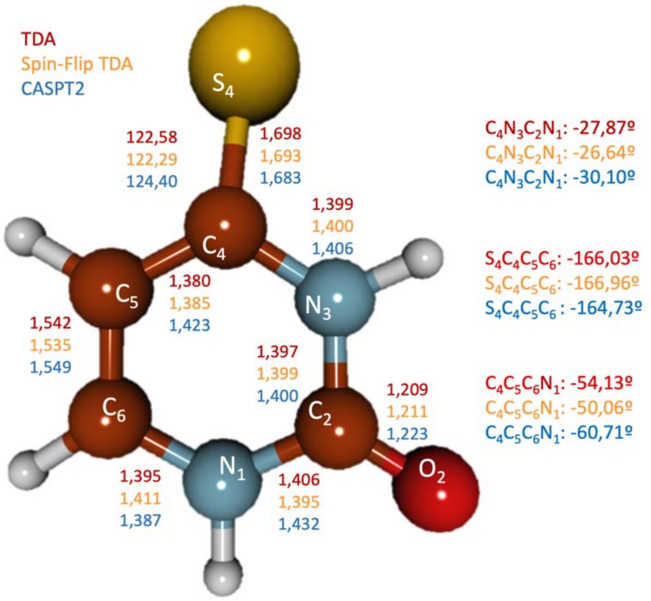

Figure S7. Most relevant geometrical parameters for the T_1S_0 ($^3\pi_S\pi_1$*/GS) singlet-triplet crossing structure of 4TU optimized at different levels of theory. Bond lengths are expressed in Å and angles and dihedrals in degrees (º).

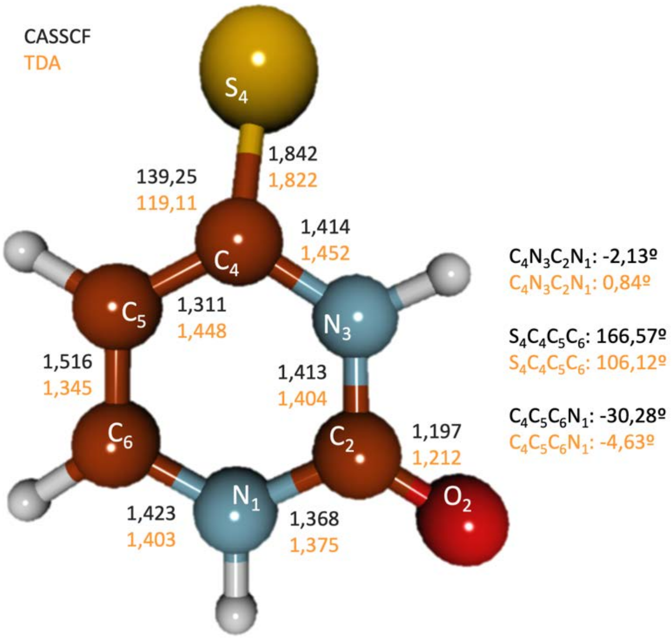

Figure S8. 4-Thiouracil T_2S_0 ($^3n_S\pi_1$*/GS) singlet-triplet crossing point geometrical parameters optimized at different levels of theory. Bond lengths are expressed in Å and angles and dihedrals in degrees (º).

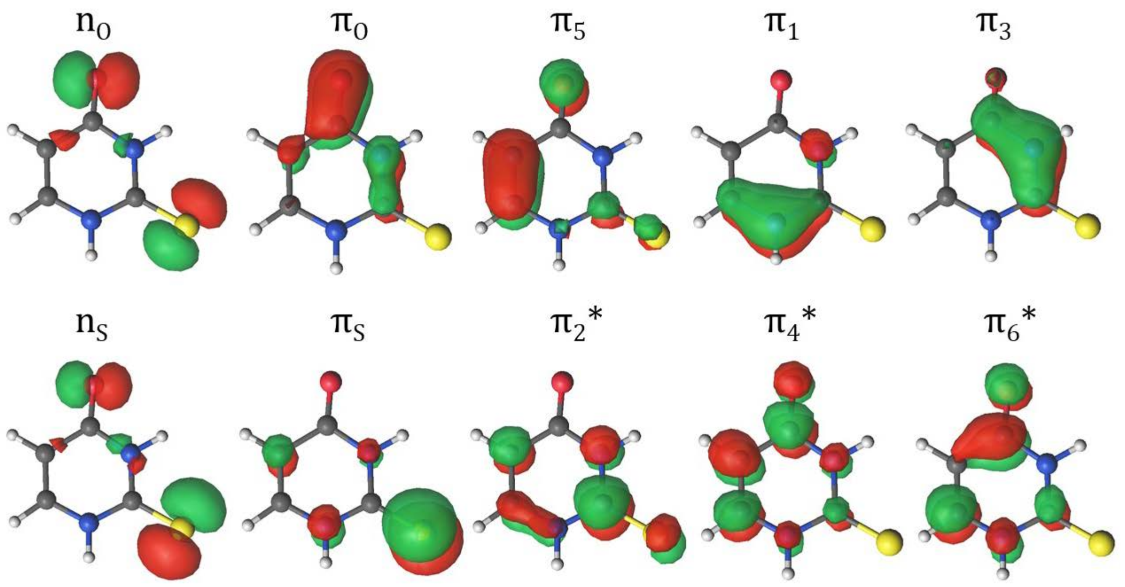

Figure S9. State-averaged valence natural orbitals computed at the SA(7)-CASSCF(14,10)/ANO-S-VDZP level of theory at the ground-state equilibrium geometry for 2-thiouracil.

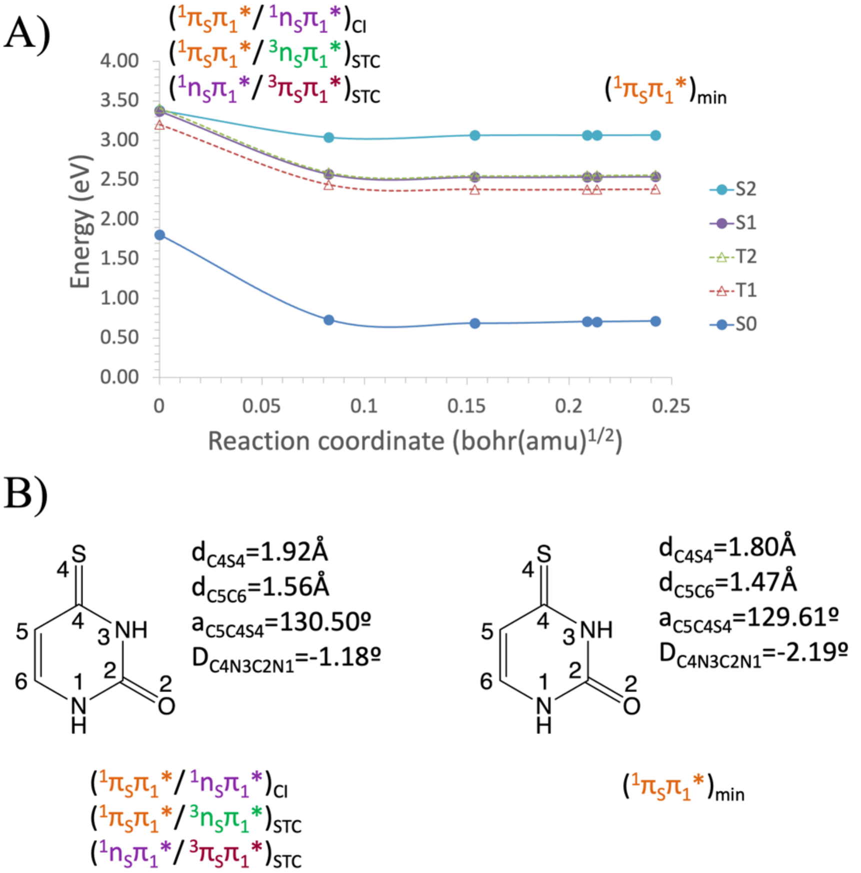

Figure S10. A) Energies of the ground and lowest-lying singlet and triplet electronic excited states of 4-thiouracil along the minimum energy path (MEP) of the S_2 $^1(\pi_S\pi_1^*)$ state from the S_2S_1 $(^1\pi_S\pi_1^*/^1n_S\pi_1^*)$ conical intersection toward the S_2 $^1(\pi_S\pi_1^*)$ equilibrium geometry, computed at the CASPT2//CASSCF(14,10)/ANO-S-VDZP level of theory. B) Main geometrical changes related with the MEP coordinate.

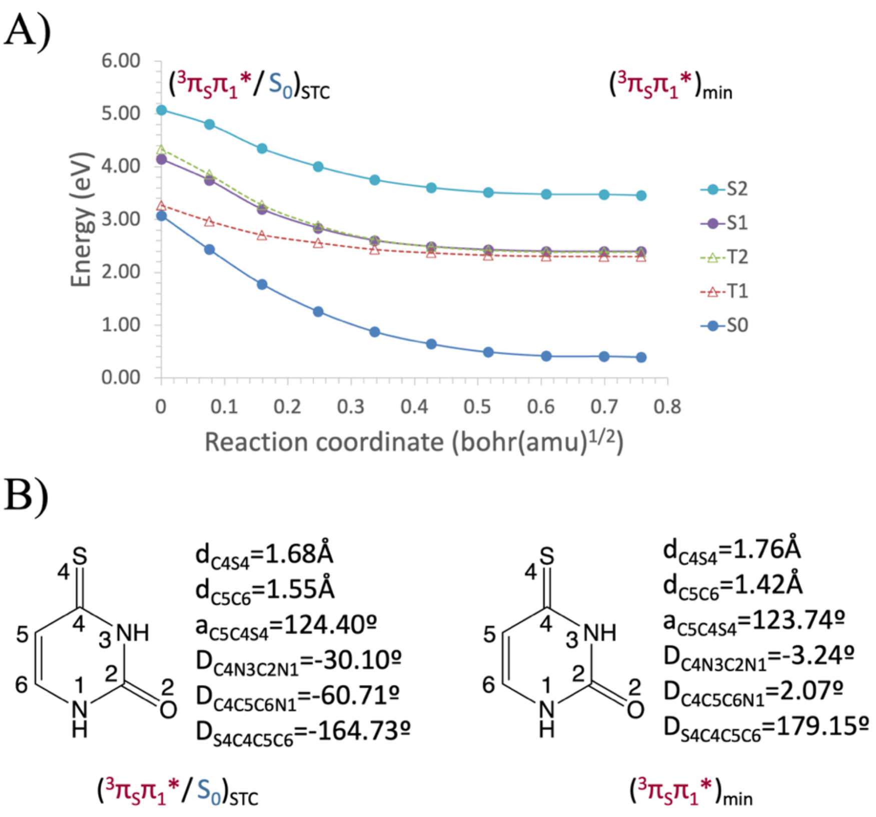

Figure S11. A) Energies of the ground and lowest-lying singlet and triplet electronic excited states of 4-thiouracil along the minimum energy path (MEP) of the $T_1\ ^3(\pi_S\pi_1^*)$ state from the T_1S_0 ($^3\pi_S\pi_1^*$/GS) singlet-triplet crossing toward the $T_1\ ^3(\pi_S\pi_1^*)$ equilibrium geometry, computed at the CASPT2//CASSCF(14,10)/ANO-S-VDZP level of theory. B) Main geometrical changes related with the MEP coordinate.

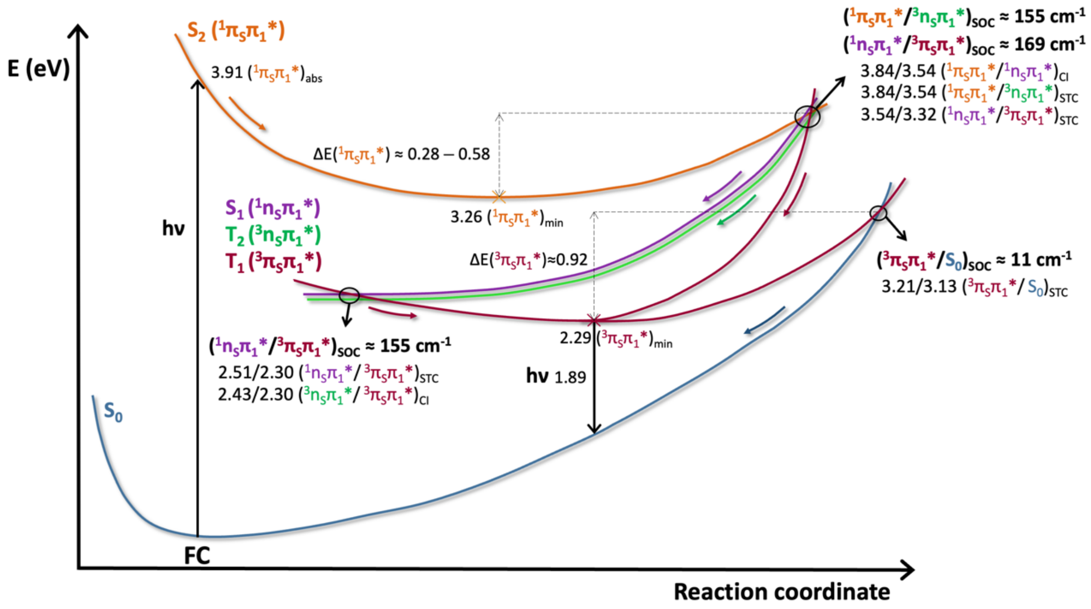

Figure S12. Schematic representation of the deactivation pathways for photoexcited 4-thiouracil based on the energy values obtained at MS-CASPT2//SA(7)-CASSCF(14,10)/ANO-S-VDZP level of theory. Main energy values (eV) of the vertical absorption, excited state minima and minimum energy crossing points, energy barriers and spin-orbit couplings (cm^{-1}) are also indicated in the figure.

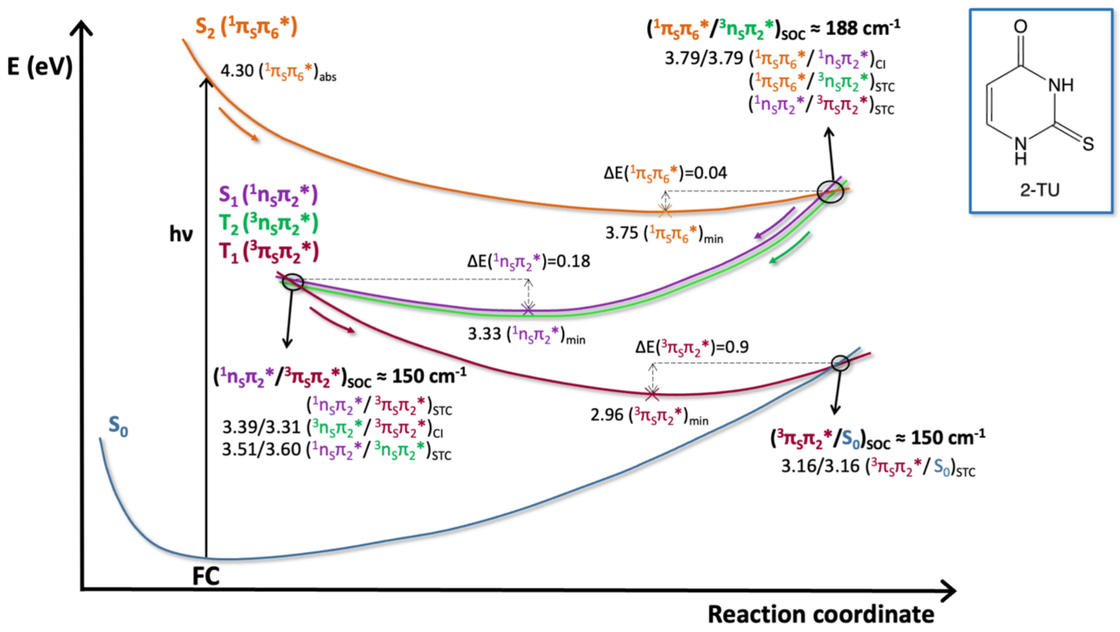

Figure S13. Schematic representation of the deactivation pathways for 2-TU. Data compiled from refs. 36 and 60, obtained at SA(4/3)-CASSCF(12,9) + MS(3/3)-CASPT2/cc-pVDZ, IPEA = 0.00 au for minima optimizations and single-point calculations, and CASSCF(14,10)/def2-svp and MRCIS(6,5)/cc-pvdz for crossing points optimization. See references for more information on the details of the paths and the methodology employed for the calculations.

Table S1. Comparison of CASPT2(14,10) energies (eV) in Franck-Condon region for 4-Thiouracil using ANO-S-VDZP, ANO-L-VDZP and ANO-L-VTZP basis sets. All energies are relative to its respective ground state minimum energy.

ANO-S-VDZP		ANO-L-VDZP		ANO-L-VTZP	
State	E	State	E	State	E
S_0	0.00	S_0	0.00	S_0	0.00
T_1	2.59	T_1	2.61	T_1	2.57
T_2	2.62	T_2	2.64	T_2	2.60
S_1	2.75	S_1	2.74	S_1	2.69
T_3	3.84	T_3	3.86	T_3	3.80
S_2	4.00	S_2	4.01	S_2	3.93
S_3	4.41	S_3	4.40	S_3	4.32
T_4	4.56	T_6	4.59	T_6	4.48
S_5	4.90	S_5	4.70	S_5	4.70
T_5	4.93	T_4	4.94	T_4	4.88
S_4	5.10	S_4	4.97	S_4	4.91
T_6	5.19	T_5	5.22	T_5	5.12
S_6	6.05	T_7	6.21	T_7	6.09
T_7	6.19	S_6	6.36	S_6	6.23

Table S2. Comparison of CASPT2(14,10) energies (eV) on the $S_2S_1T_2T_1$ MECP for 4-Thiouracil using ANO-S-VDZP, ANO-L-VDZP and ANO-L-VTZP basis sets. All energies are relative to its respective ground state minimum energy.

ANO-S-VDZP		ANO-L-VDZP		ANO-L-VTZP	
State	E	State	E	State	E
S_0	1.80	S_0	1.87	S_0	1.87
T_1	3.20	T_1	3.24	T_1	3.26
S_1	3.37	T_2	3.44	S_1	3.42
S_2	3.38	S_1	3.46	S_2	3.45
T_2	3.41	S_2	3.59	T_2	3.46
T_3	4.91	T_3	4.95	T_3	4.91
S_5	5.07	S_3	5.43	S_5	5.13
S_4	5.29	T_5	5.55	S_4	5.36
T_5	5.51	S_5	5.61	T_5	5.51
S_3	5.72	S_4	5.96	S_3	5.73
T_4	5.93	T_4	5.97	T_4	5.94
T_7	6.48	T_7	6.52	T_7	6.50
S_6	6.83	S_6	7.01	S_6	6.81
T_6	8.06	T_6	8.10	T_6	8.05

Table S3. Comparison of CASPT2(14,10) spin-orbit coupling (cm^{-1}) between S_1T_1 and S_2T_2 on the $S_2S_1T_2T_1$ MECP for 4-Thiouracil using ANO-S-VDZP, ANO-L-VDZP and ANO-L-VTZP basis sets.

	ANO-S-VDZP	ANO-L-VDZP	ANO-L-VTZP
S_1T_1	169	166	167
S_2T_2	107	120	109

Table S4. Comparison of CASPT2(14,10) energies (eV) on the $S_1T_2T_1$ MECP for 4-Thiouracil using ANO-S-VDZP, ANO-L-VDZP and ANO-L-VTZP basis sets. All energies are relative to its respective ground state minimum energy.

ANO-S-VDZP		ANO-L-VDZP		ANO-L-VTZP	
State	E	State	E	State	E
S_0	0.31	S_0	0.39	S_0	0.47
T_1	2.24	T_1	2.26	T_1	2.34
T_2	2.31	T_2	2.35	T_2	2.41
S_1	2.33	S_1	2.43	S_1	2.48
S_2	3.36	S_2	3.46	S_2	3.47
T_3	3.97	T_3	3.91	T_3	4.03
S_4	4.21	S_3	4.34	S_4	4.51
S_5	4.34	S_5	4.78	S_5	4.73
S_3	4.80	T_4	4.97	S_3	4.90
T_4	4.94	S_4	5.00	T_4	4.99
T_6	5.01	T_5	5.12	T_6	5.07
T_5	5.24	T_6	5.22	T_5	5.25
S_6	5.52	T_7	5.60	S_6	6.00
T_7	7.22	S_6	5.78	T_7	7.26

Table S5. Comparison of CASPT2(14,10) spin-orbit coupling (cm^{-1}) between S_1T_1 on the $S_1T_2T_1$ MECP for 4-Thiouracil using ANO-S-VDZP, ANO-L-VDZP and ANO-L-VTZP basis sets.

	ANO-S-VDZP	ANO-L-VDZP	ANO-L-VTZP
S_1T_1	155	160	162

Table S6. Reference weights for all roots of the 6 main structures for SS-CASPT2 calculations. Reference weight obtained with the formula $w = (1 + \alpha)^{-\frac{N}{2}}$, where N represents the number of correlated electrons (excluding those in the frozen core orbitals), and α is a constant of approximately 0.015, is $w = 0.731$.

	FC region	S_2 minimum	$S_2S_1T_2T_1$ MECP	$S_1T_2T_1$ MECP	T_1 minimum	S_0T_1 MECP
S_0	0.7231	0.72054	0.71348	0.72222	0.72203	0.71485
S_1	0.72126	0.71969	0.72103	0.7229	0.723	0.71455
S_2	0.70929	0.70774	0.70302	0.70391	0.70595	0.70692
S_3	0.69976	0.69764	0.70858	0.71128	0.71189	0.68898
S_4	0.70932	0.70806	0.68877	0.69521	0.69544	0.70675
S_5	0.68721	0.68572	0.67866	0.68288	0.68388	0.67986
S_6	0.70155	0.69981	0.68785	0.68339	0.68545	0.66871
T_1	0.72364	0.72229	0.72365	0.72444	0.72446	0.71647
T_2	0.7237	0.72241	0.72405	0.72462	0.72475	0.7189
T_3	0.71056	0.70834	0.70282	0.71238	0.71189	0.71149
T_4	0.71255	0.71158	0.7125	0.71569	0.71545	0.71453
T_5	0.70345	0.70208	0.69787	0.70413	0.70426	0.71544
T_6	0.69552	0.69242	0.71537	0.68795	0.68971	0.71673
T_7	0.70104	0.69697	0.68449	0.71636	0.71653	0.69169

Table S7. Comparison of CASPT2(14,10) energies (eV) on the Franck-Condon region and S_2 and T_1 minima for 4-Thiouracil using SS and MS-CASPT2. All energies are relative to its respective ground state minimum energy.

FC region				S_2 minimum				T_1 minimum			
SS-CASPT2		MS-CASPT2		SS-CASPT2		MS-CASPT2		SS-CASPT2		MS-CASPT2	
State	E (eV)	State	E (eV)	State	E (eV)	State	E (eV)	State	E (eV)	State	E (eV)
S_0	0.00	S_0	0.00	S_0	0.63	S_0	0.75	S_0	0.23	S_0	0.41
T_1	2.59	T_1	2.66	T_1	2.31	T_1	2.43	T_1	2.19	T_1	2.29
T_2	2.62	T_2	2.74	T_2	2.51	T_2	2.63	T_2	2.34	T_2	2.46
S_1	2.75	S_1	2.86	S_1	2.56	S_1	2.68	S_1	2.34	S_1	2.53
T_3	3.84	T_3	3.85	S_2	3.14	S_2	3.26	S_2	3.22	S_2	3.42
S_2	4.00	S_2	3.91	T_3	3.89	T_3	4.01	T_3	3.82	T_3	3.84
S_3	4.41	S_3	4.46	S_3	4.49	S_3	4.61	S_4	4.13	S_3	4.32
T_4	4.56	T_4	4.73	S_4	4.78	S_5	4.90	S_5	4.33	S_5	4.88
S_5	4.90	T_5	5.04	T_4	4.82	T_5	4.94	S_3	4.78	T_4	5.01
T_5	4.93	S_4	5.22	T_5	4.96	T_4	5.08	T_4	4.89	T_5	5.05
S_4	5.10	T_6	5.40	S_5	5.05	S_4	5.17	T_6	5.01	S_4	5.08
T_6	5.19	S_5	5.42	T_6	5.40	T_6	5.52	T_5	5.03	T_6	5.27
S_6	6.05	S_6	6.17	T_7	5.97	T_7	6.09	S_6	5.56	T_7	5.76
T_7	6.19	T_7	6.32	S_6	6.04	S_6	6.16	T_7	7.18	S_6	5.86
f ($S_0 \rightarrow S_2$)	0.419		0.640								

Table S8. Comparison of CASPT2(14,10) energies (eV) on the minimum energy crossing points for 4-Thiouracil using SS and MS-CASPT2. All energies are relative to its respective ground state minimum energy.

$S_2S_1T_2T_1$ MECP				$S_1T_2T_1$ MECP				S_0T_1 MECP			
SS-CASPT2		MS-CASPT2		SS-CASPT2		MS-CASPT2		SS-CASPT2		MS-CASPT2	
State	E (eV)	State	E (eV)	State	E (eV)	State	E (eV)	State	E (eV)	State	E (eV)
S_0	1.80	S_0	1.61	S_0	0.31	S_0	0.27	S_0	2.99	S_0	3.13
T_1	3.20	T_1	3.32	T_1	2.24	T_1	2.30	T_1	3.17	T_1	3.21
S_1	3.37	T_2	3.54	T_2	2.31	T_2	2.43	S_1	4.17	T_2	4.29
S_2	3.38	S_1	3.54	S_1	2.33	S_1	2.51	T_2	4.32	S_1	4.30
T_2	3.41	S_2	3.84	S_2	3.36	S_2	3.40	S_3	4.62	S_3	4.72
T_3	4.91	T_3	4.78	T_3	3.97	T_3	3.59	S_2	4.94	S_2	5.10
S_5	5.07	S_3	5.22	S_4	4.21	S_3	4.20	T_3	5.21	T_3	5.26
S_4	5.29	S_4	6.04	S_5	4.34	T_4	4.95	S_5	5.83	S_5	5.94
T_5	5.51	T_4	6.06	S_3	4.80	T_5	5.05	S_6	6.29	T_4	6.59
S_3	5.72	S_5	6.12	T_4	4.94	S_4	5.14	S_4	6.50	S_4	6.62
T_4	5.93	T_5	6.15	T_6	5.01	S_5	5.36	T_4	6.61	S_6	6.74
T_7	6.48	T_6	6.68	T_5	5.24	T_6	5.69	T_5	6.85	T_5	6.84
S_6	6.83	S_6	7.08	S_6	5.52	S_6	5.86	T_6	7.11	T_6	7.12
T_6	8.06	T_7	8.27	T_7	7.22	T_7	5.97	T_7	7.25	T_7	7.48

Table S9. Absolute (a.u.) and relative (eV) energies at CASSCF(14,10)/ANO-S-VDZP level of the optimized minima and minimum energy crossing points along the deactivation path of 4-thiouracil. All energies are relative to the CASSCF S_0 energy at the Franck-Condon region.

	$S_{0,min}$ Franck Condon		$S_{2,min}$ Last point MEP S_2		$T_{1,min}$ Last point MEP T_1		$S_2S_1T_2T_1$ MECP		$S_1T_2T_1$ MECP		S_0T_1 MECP	
	E_{CASSCF} (a.u.)	E_{CASSCF} (eV)	E_{CASSCF} (a.u.)	E_{CASSCF} (eV)	E_{CASSCF} (a.u.)	E_{CASSCF} (eV)	E_{CASSCF} (a.u.)	E_{CASSCF} (eV)	E_{CASSCF} (a.u.)	E_{CASSCF} (eV)	E_{CASSCF} (a.u.)	E_{CASSCF} (eV)
S_0	-735.2509	0.00	-735.2162	0.94	-735.2316	0.53	-735.1597	2.48	-735.2299	0.57	-735.1227	3.49
S_1	-735.1417	2.97	-735.1476	2.81	-735.1588	2.51	-735.1183	3.61	-735.1598	2.48	-735.0784	4.70
S_2	-735.0652	5.06	-735.1053	3.96	-735.0965	4.20	-735.0866	4.47	-735.0885	4.42	-735.0395	5.75
S_3	-735.0425	5.67	-735.0389	5.77	-735.0496	5.48	-735.0066	6.65	-735.0494	5.48	-734.9990	6.86
S_4	-735.0313	5.97	-735.0354	5.87	-735.0447	5.61	-734.9968	6.91	-735.0437	5.64	-734.9847	7.25
S_5	-735.0078	6.62	-735.0066	6.65	-735.0125	6.49	-734.9756	7.49	-735.0100	6.56	-734.9371	8.54
S_6	-734.9883	7.15	-734.9828	7.29	-734.9573	7.99	-734.9218	8.96	-734.9542	8.07	-734.9149	9.14
T_1	-735.1487	2.78	-735.1601	2.47	-735.1708	2.18	-735.1333	3.20	-735.1692	2.22	-735.1233	3.47
T_2	-735.1478	2.81	-735.1535	2.65	-735.1659	2.31	-735.1267	3.38	-735.1671	2.28	-735.0846	4.53
T_3	-735.0908	4.36	-735.0783	4.70	-735.0898	4.39	-735.0350	5.88	-735.0864	4.48	-735.0423	5.68
T_4	-735.0384	5.78	-735.0431	5.65	-735.0563	5.29	-735.0119	6.50	-735.0564	5.29	-734.9982	6.88
T_5	-735.0221	6.23	-735.0302	6.00	-735.0308	5.99	-735.0025	6.76	-735.0248	6.15	-734.9919	7.05
T_6	-735.0120	6.50	-734.9870	7.18	-734.9951	6.96	-734.9442	8.35	-734.9922	7.04	-734.9852	7.23
T_7	-735.0052	6.69	-734.9780	7.43	-734.9775	7.44	-734.9378	8.52	-734.9768	7.46	-734.9283	8.78

Table S10. Absolute (a.u.) and relative (eV) energies at CASPT2(14,10)/ANO-S-VDZP level of the optimized minima and minimum energy crossing points along the deactivation path of 4-thiouracil. All energies are relative to the CASPT2 S_0 energy at the Franck-Condon region.

	$S_{0,min}$ Franck Condon		$S_{2,min}$ Last point MEP S_2		$T_{1,min}$ Last point MEP T_1		$S_2S_1T_2T_1$ MECP		$S_1T_2T_1$ MECP		S_0T_1 MECP	
	E_{CASPT2} (a.u.)	E_{CASPT2} (eV)	E_{CASPT2} (a.u.)	E_{CASPT2} (eV)	E_{CASPT2} (a.u.)	E_{CASPT2} (eV)	E_{CASPT2} (a.u.)	E_{CASPT2} (eV)	E_{CASPT2} (a.u.)	E_{CASPT2} (eV)	E_{CASPT2} (a.u.)	E_{CASPT2} (eV)
S_0	-736.3293	0.00	-736.3061	0.63	-736.3207	0.23	-736.2630	1.80	-736.3180	0.31	-736.2192	2.99
S_1	-736.2283	2.75	-736.2353	2.56	-736.2431	2.34	-736.2056	3.37	-736.2436	2.33	-736.1760	4.17
S_2	-736.1821	4.00	-736.2140	3.14	-736.2109	3.22	-736.2051	3.38	-736.2057	3.36	-736.1478	4.94
S_3	-736.1674	4.41	-736.1644	4.49	-736.1537	4.78	-736.1191	5.72	-736.1528	4.80	-736.1594	4.62
S_4	-736.1420	5.10	-736.1536	4.78	-736.1774	4.13	-736.1350	5.29	-736.1745	4.21	-736.0903	6.50
S_5	-736.1493	4.90	-736.1438	5.05	-736.1701	4.33	-736.1428	5.07	-736.1699	4.34	-736.1151	5.83
S_6	-736.1071	6.05	-736.1074	6.04	-736.1249	5.56	-736.0783	6.83	-736.1264	5.52	-736.0981	6.29
T_1	-736.2342	2.59	-736.2442	2.31	-736.2490	2.19	-736.2116	3.20	-736.2468	2.24	-736.2128	3.17
T_2	-736.2330	2.62	-736.2372	2.51	-736.2434	2.34	-736.2041	3.41	-736.2442	2.31	-736.1705	4.32
T_3	-736.1883	3.84	-736.1863	3.89	-736.1887	3.82	-736.1490	4.91	-736.1834	3.97	-736.1378	5.21
T_4	-736.1618	4.56	-736.1521	4.82	-736.1496	4.89	-736.1113	5.93	-736.1479	4.94	-736.0863	6.61
T_5	-736.1482	4.93	-736.1471	4.96	-736.1444	5.03	-736.1269	5.51	-736.1366	5.24	-736.0777	6.85
T_6	-736.1387	5.19	-736.1308	5.40	-736.1453	5.01	-736.0333	8.06	-736.1453	5.01	-736.0680	7.11
T_7	-736.1017	6.19	-736.1100	5.97	-736.0653	7.18	-736.0912	6.48	-736.0638	7.22	-736.0629	7.25

Table S11. CASPT2(14,10)//CASSCF(14,10)/ANO-L-VTZP energies (eV) at the equilibrium structure of S_1, S_2 and T_1 states of 4-thiouracil and of S_1 and S_2 states of 2-thiouracil. All energies are relative to its respective ground state minimum energy.

	4-thiouracil				2-thiouracil			
	E_{S1min} (planar)	E_{S2min} (planar)	E_{T1min} (planar)	E_{S2min} (pyramidal)	E_{S2min} (planar)	E_{S2min} (pyramidal)	E_{S1min} (planar)	E_{S1min} (pyramidal)
S_0	0.33	0.74	0.28	2.19	0.81	2.37	0.21	1.44
S_1	2.44	2.64	2.44	3.25	3.67	3.52	3.35	3.20
S_2	3.38	3.16	3.40	3.79	3.65	3.67	3.89	3.71
S_3	4.91	4.96	4.89	5.55	4.52	5.66	4.46	5.36
S_4	4.33	4.60	4.31	5.80	4.86	5.84	4.83	5.61
S_5	4.50	4.51	4.49	5.78	5.01	5.72	4.75	4.75
S_6	5.66	5.90	5.65	6.62	6.95	6.78	5.64	6.49
T_1	2.32	2.50	2.32	3.09	3.20	3.23	3.04	3.05
T_2	2.43	2.66	2.42	3.14	3.70	3.35	3.34	3.07
T_3	3.97	4.11	3.95	5.18	3.90	5.62	3.82	4.93
T_4	5.01	5.09	4.98	5.62	4.24	6.38	4.68	5.83
T_5	5.06	4.93	5.05	5.74	4.46	5.99	4.88	5.41
T_6	5.16	5.50	5.11	6.33	5.89	6.32	4.65	5.49
T_7	7.33	7.36	7.31	6.69	6.77	6.66	6.23	6.17

Cartesian coordinates

12

Min S_0, Franck Condon

H	-2.12375413	-1.10436047	-0.00000000
C	-2.14434675	2.68166232	0.00000000
N	-1.00891903	1.91060930	0.00000000
C	-0.93517683	0.52968247	0.00000000
N	-2.15094791	-0.10585433	0.00000000
C	-3.35093089	0.57740690	-0.00000000
C	-3.39172863	1.92519862	0.00000000
S	-2.07166381	4.32456061	-0.00000000
H	-0.12377763	2.38154361	0.00000000
O	0.11073714	-0.05281739	-0.00000000
H	-4.23801136	-0.03606977	-0.00000000
H	-4.32323418	2.46393713	0.00000000

12

$S_{2,min}$, Last point MEP S_2

H	-2.0605021	-1.1197809	-0.0018945
C	-2.2380456	2.5906721	0.0004721
N	-1.0474500	1.9176814	0.0006085
C	-0.9313404	0.5355742	0.0000246
N	-2.1398467	-0.1208026	-0.0008173
C	-3.3750468	0.4652042	-0.0002278
C	-3.4143379	1.9202446	0.0009140
S	-2.0125807	4.3986648	-0.0004383
H	-0.1940498	2.4439119	0.0009763
O	0.1210127	-0.0350968	0.0001545
H	-4.2325063	-0.1810037	-0.0007378
H	-4.3517010	2.4474693	0.0019240

12

MECP $S_2S_1T_2T_1$

H	-2.0400031	-1.1079873	-0.0339229
C	-2.2559520	2.5535430	0.0089671
N	-1.0705385	1.9108443	0.0213410
C	-0.9337467	0.5443859	0.0005278
N	-2.1450870	-0.1182191	-0.0086424
C	-3.3823580	0.3982651	0.0129644
C	-3.4399559	1.9551390	0.0010272
S	-1.8010779	4.4190073	-0.0270577
H	-0.3123971	2.5616895	0.0333946
O	0.0939491	-0.0455400	-0.0199806
H	-4.2112308	-0.2730221	0.0334783
H	-4.3779968	2.4646328	-0.0211377

12

MECP $S_1T_1T_2$

H	-2.1461393	-1.1046084	0.0323851
C	-2.1217264	2.6288245	-0.1186258

N	-0.9414478	1.8777870	0.0501142
C	-0.9307760	0.4928172	0.0104628
N	-2.1606972	-0.1083477	0.0938017
C	-3.3731853	0.5865598	0.0180444
C	-3.3525976	1.9716331	-0.0774294
S	-1.9423517	4.3823050	0.0170327
H	-0.0707005	2.2771148	-0.2395391
O	0.0904374	-0.1264723	-0.0594878
H	-4.2713790	-0.0045679	0.0227804
H	-4.2756182	2.5275922	-0.1240879

12

$T_{1,min}$, Last point MEP T_1

H	-2.1375950	-1.1099809	0.0271330
C	-2.1295365	2.6335334	-0.0235263
N	-0.9534315	1.8682982	-0.0256837
C	-0.9260057	0.4941313	-0.0038569
N	-2.1605734	-0.1116768	0.0137144
C	-3.3787441	0.5688601	0.0061558
C	-3.3469979	1.9877201	-0.0085671
S	-1.9396505	4.3851932	-0.0064062
H	-0.0640857	2.3227005	-0.0350136
O	0.0961327	-0.1275556	0.0016771
H	-4.2753540	-0.0216031	0.0059377
H	-4.2623907	2.5555639	-0.0079603

12

MECP S_0T_1

H	2.6032411	1.0104147	0.6155594
C	-0.9227938	0.0623080	-0.0702611
N	0.1035077	-0.8420403	-0.3960573
C	1.3979672	-0.5890399	0.0721479
N	1.6704163	0.8089065	0.2201908
C	0.9413932	1.6627571	-0.5942388
C	-0.4599564	1.4051728	0.0122533
S	-2.5209461	-0.4091742	0.1683262
H	-0.1362983	-1.8355813	-0.5085381
O	2.2363563	-1.4441609	0.3182885
H	1.3443306	2.6787765	-0.7032377
H	-0.8170205	2.0318958	0.8457186

12

$S_{1,min}$, Optimized from last point MEP S_1

H	-2.14145874	-1.10993165	0.02424732
C	-2.13795254	2.62647913	-0.02677620
N	-0.95531751	1.87172261	-0.02611763
C	-0.92859571	0.49336429	-0.00278894
N	-2.15926109	-0.11208802	0.01347675
C	-3.37511874	0.58042028	0.00567118
C	-3.35570696	1.97169102	-0.00774641

S	-1.94109042	4.38534596	-0.00820894
H	-0.05790723	2.30877957	-0.03875343
O	0.10131908	-0.12287990	0.00122696
H	-4.27145809	-0.01217594	0.01016245
H	-4.28064645	2.52644365	-0.00889232

12

$S_{2,min}$, Optimized from last point MEP S_2

H	-2.05782790	-1.12296045	-0.00101294
C	-2.24489111	2.59503363	0.00068896
N	-1.06263882	1.91934828	0.00034652
C	-0.93707431	0.53809506	-0.00018148
N	-2.13835021	-0.12397391	-0.00049086
C	-3.37284114	0.46277841	-0.00008411
C	-3.42096108	1.91938603	0.00054237
S	-1.96389744	4.39207165	0.00100427
H	-0.20969683	2.44639865	0.00041234
O	0.12457996	-0.01679769	-0.00041808
H	-4.22950388	-0.18448277	-0.00046182
H	-4.36329185	2.43784160	0.00061393

12

$T_{1,min}$, Optimized from last point MEP T_1

H	-2.14124154	-1.10996373	0.00089440
C	-2.13348528	2.62884629	-0.00473149
N	-0.95813160	1.86997833	0.00336112
C	-0.92669709	0.49460112	-0.00171700
N	-2.16192463	-0.11137628	0.00272643
C	-3.37446411	0.57090853	0.00605521
C	-3.34461731	1.98416679	-0.00278075
S	-1.95063809	4.38513645	-0.01831609
H	-0.07035040	2.32814692	-0.00625941
O	0.09818408	-0.12942221	-0.00801045
H	-4.27221946	-0.01870992	0.00692104
H	-4.26143858	2.55005731	-0.00521320

12

$S_{1,min}$, Optimized with ANO-L-VTZP basis set

H	-2.10986621	-1.07709525	0.00014469
C	-2.16995250	2.64682005	0.00007419
N	-0.98376889	1.89776454	0.00005669
C	-0.93088048	0.53710888	-0.00005393
N	-2.14553932	-0.08799615	0.00000249
C	-3.36207370	0.58135326	-0.00001267
C	-3.41613432	1.90766351	0.00006285
S	-2.04965957	4.42842513	-0.00014839
H	-0.09851439	2.34230045	0.00009098
O	0.09943004	-0.06684183	-0.00013489
H	-4.23325300	-0.03863610	-0.00015440
H	-4.35154167	2.42463252	0.00007239

12

$S_{2,min}$, planar. Optimized with ANO-L-VTZP basis set

H	-2.08695569	-1.09711771	0.00000486
C	-2.22854663	2.58724304	-0.00000149
N	-1.03262239	1.90087827	-0.00000431
C	-0.94153158	0.52296860	-0.00000056
N	-2.14407077	-0.10659979	0.00000097
C	-3.37621187	0.51573577	-0.00000324
C	-3.39258656	1.94525851	-0.00000224
S	-1.91136880	4.48052880	0.00000831
H	-0.18364926	2.41554021	-0.00000420
O	0.10474642	-0.04317581	0.00000217
H	-4.24180838	-0.10480924	0.00000037
H	-4.31714849	2.47904835	-0.00000064

12

$S_{2,min}$, distorted. Optimized with ANO-L-VTZP basis set

H	-2.10504166	-1.11631222	-0.01868961
C	-2.22904683	2.61146519	-0.03952313
N	-1.08474586	1.90903424	0.00870307
C	-0.95807991	0.53426576	0.01703469
N	-2.15861669	-0.12609520	-0.01877370
C	-3.36574660	0.50995893	-0.03487921
C	-3.43848533	1.86048056	-0.03544993
S	-2.20953900	4.12760125	1.32592030
H	-0.22928680	2.41349187	0.07250763
O	0.09683525	-0.02074997	0.04489139
H	-4.22697583	-0.12428393	-0.07183915
H	-4.37528150	2.37081484	-0.02831695

12

$T_{1,min}$, Optimized with ANO-L-VTZP basis set

H	-2.10226700	-1.08727297	-0.00000004
C	-2.19011510	2.63088475	0.00000011
N	-0.99716589	1.90583622	0.00000015
C	-0.93367899	0.53475432	-0.00000001
N	-2.14346970	-0.09819747	-0.00000001
C	-3.37072886	0.56239836	0.00000001
C	-3.38908261	1.94115857	0.00000001
S	-2.04961837	4.37676346	-0.00000021
H	-0.11884683	2.36423716	0.00000015
O	0.10949179	-0.05400594	-0.00000017
H	-4.24569333	-0.04780674	0.00000005
H	-4.32057912	2.46674927	-0.00000004
